# Modeling impacts of climate-induced yield variability and adaptations on wheat and maize in a sub-tropical monsoon climate - using fuzzy logic

**DOI:** 10.1038/s41598-025-09820-3

**Published:** 2025-07-16

**Authors:** Md. Abdul Kaium, Md. Sharif Ahmed, Muhammad Habib-Ur-Rahman, Md. Saidul Islam, Yeasmin Akter Ratry, Md Mostofa Uddin Helal, Muhammad Ali Fardoush Siddquy, Most. Moslema Haque, Ahsan Raza, Fatma Mansour, Majed Alotaibi, Ayman El Sabagh, Reimund P. Roetter

**Affiliations:** 1 Department of Crop Science and Technology, University of Rajshahi, Rajshahi, 6205 Bangladesh; 2 Institute of Natural Resources Research and Development, Rajshahi, 6206 Bangladesh; 3 Department of Crop Sciences, Tropical Plant Production and Agricultural Systems Modelling (TROPAGS), University of Goettingen, Grisebachstr. 6, 37077 Goettingen, Germany; 4 Department of Mathematics, University of Rajshahi, Rajshahi, 6205 Bangladesh; 5 State Key Laboratory of Sustainable Dryland Agriculture, Institute of Wheat Research, College of Agriculture, Shanxi Agricultural University, Linfen, 041000 Shanxi China; 6 Graduate School of Bioresources, Mie University, 1577 Kurimamachiya-Cho, Tsu, 514- 8507 Mie Japan; 7 Leibniz Centre for Agricultural Landscape Research (ZALF), Eberswalder Straße 84, 15374 Müncheberg, Germany; 8 Department of Economics, Business and Economics Faculty, Siirt University, Siirt, Turkey; 9 Plant Production Department, College of Food and Agricultural Sciences, King Saud University, P.O. Box. 2460, Riyadh, 11451 Saudi Arabia; 10 Faculty of Agriculture, Department of Field Crops, Siirt University, Siirt, 56100 Turkey; 11 Campus Centre of Biodiversity and Sustainable Land Use (CBL), University of Goettingen, Buesgenweg 1, 37077 Goettingen, Germany

**Keywords:** Adaptation, elevated temperature, Phenological development, Rainfall variability, Sub-tropical monsoon climate, Plant sciences, Environmental sciences

## Abstract

Climate change is causing more frequent and extraordinary extreme weather events that are already negatively affecting crop production. There is a need for improved climate risk assessment by developing smart adaptation strategies for sustainable future crop production. This study aims to assess yield impacts of extreme temperatures and rainfall variability on wheat, and winter and summer season-planted maize in northwestern Bangladesh. Utilizing a machine learning approach, future yield patterns were predicted for these crops under various climate change scenarios. Additionally, the study developed adaptation strategies focusing on prediction of optimum sowing windows for wheat and maize to minimize climate risk-related yield losses jeopardizing food security. A fuzzy logical model was applied, incorporating a set of fuzzy rules to estimate the probable yields of wheat and maize (winter and summer growing seasons). Key climatic variables (temperature and rainfall) were added as model inputs, enabling the model to handle uncertainty and nonlinear interactions in the climate–yield relationship. Findings demonstrated that climate change has significant negative impacts at the different phenological stages of both wheat and maize (winter and summer seasons), with yield levels generally showing notable declines. Only small variations in optimal temperature and rainfall patterns affected crop yields significantly. Moreover, maize summer yield was consistently lower than maize winter as the temperature prevails high during the maize summer season (April to July). The study found that the wheat crop, maize winter, and maize summer have as optimal planting windows November 1–7, November 1–10, and February 20 - March 7, respectively. Such adaptation would ensure maximum yield and effective reduction of climate change risks. Outcomes of this study contribute to multiple Sustainable Development Goals (SDGs), especially three; zero hunger (SDG2), climate action (SDG13), and life on land (SDG14). These adaptations identified in this study can support policymakers and stakeholders to combat the impact of extreme climate – and achieving optimal yield. The approach is also applicable to other regions of the country and similar monsoon climates.

**Supplementary Information:**

The online version contains supplementary material available at 10.1038/s41598-025-09820-3.

## Introduction

Climate change poses significant threats to global food security, emerging as one of the biggest challenges of the twenty-first century^1–5^. The necessity to feed an ever-growing population while preserving fragile ecosystems is becoming increasingly challenging, and associated threats are projected to intensify in future decades^6–11^. The future projections indicate that agricultural production systems would be negatively affected more in Sub-Saharan Africa, South Asia, and Southeast Asia, where farming communities are disproportionately poor and vulnerable^6,12–20^. Rising temperatures and unpredictable weather patterns disrupt traditional farming practices, leading to reduced crop yields and increased food insecurity in many regions^6,21–24^. Changes in climatic patterns such as elevated temperatures, erratic rainfall, and increasing frequency of extreme events (droughts and floods), pose serious challenges to agricultural productivity and sustainability^25–29^ These shifts in weather patterns affect crop growth cycles, water availability, soil conditions, and crop management practices, ultimately lead to reduced crop yields, and diminished food quality particularly in vulnerable regions^30–35^. Addressing these issues requires innovative solutions to ensure that the global food production and supply meets the needs of future generations. Projections indicate that the global mean temperature may rise 1.8 to 4.0 °C by the end of this century in South Asia^36^. Additionally, global agricultural productivity is projected to decrease by 3–16% by 2080 that affect food security^24,37–40^.

The global demand for food continues to rise with future projections indicating the need of production increases by ca. 70% to feed the world by 2050^41^. Food growing demand further intensifies the challenges for food production and food security, particularly in the context of climate change and limited natural resources. For instance, staple crops like wheat, rice, and maize have experienced declining productivity due to heat stress and changing precipitation patterns, particularly in vulnerable areas^12,25,32,42,43^. In almost 40 nations worldwide, wheat is regarded as the main staple crop, providing essential calories and protein to around 85% and 82% of the global population, respectively^44^. However, maize is extensively utilized by industries for feed (especially for poultry and livestock), and for food. Global wheat yield in 2021 amounted to about 220 million hectares, yielding 770 million tons of grain (3.5 t ha^-1^); in contrast, t ha^- 1^global maize yield in 2021 is estimated to be 205 million hectares, giving 1210 million tons of grain (5.90 t ha^-1^)^45,46^. In Bangladesh, however, wheat is the second most important cereals which was cultivated in 316 thousand ha and contributed 1.17 million MT of grains (3.70 t ha^- 1^) in 2022-23 (BBS, 2023). Unlike, winter and summer maize covered 1031 thousand ha and 195 thousand ha areas and produced about 4 million MT and 0.55 million MT (3.87 and 2.82 t ha^- 1^), respectively at the same time. These trends demonstrate the existing scenario of wheat and maize yield in the country and call for urgent research. Furthermore, the Northwestern part (Rajshahi division) is the most important wheat and maize-growing region in Bangladesh, contributing 36% of the total wheat yield of Bangladesh and a significant amount of maize^47–49^.

Production of maize and wheat is highly sensitive to abiotic stresses (elevated temperature and rainfall variability), these are significant factors affect the plant critical growth stages, development, and yield production processes^50–55^. Global wheat production is predicted to decline by 6% for every 1 °C increase in temperature^56^. Moreover, even a 1 °C increase in average temperature during the reproductive stage may result in a greater loss of grain yield (Poudel & Poudel, 2020). Depending on the location, the direct effects of climate change will result in losses in wheat yield of 1–8% due to variations in temperature and precipitation^57^. For Bangladesh, as one of the most vulnerable countries to the effects of climate change because of its proximity to the Bay of Bengal and its low-lying deltaic landforms; temperatures have progressively increased over the past three decades^58^. According to projected climate models, Bangladesh’s most extreme temperatures are predicted to rise from 1 °C to 4.1 °C by 2100, while the country’s lowest temperature is predicted to rise from 1 °C to 4.4 °C^59^. Wheat yield is expected to decline by 32% by 2050 due to increasing mean temperature in Bangladesh^60^. After all, wheat is more susceptible to heat stress during its reproductive phase than during vegetative phase, where optimum temperature for wheat growth and reproductive stages occurs at approximately 25 °C and between 15 and 20 °C respectively^61–63^. In contrast, the average ideal temperature for maize entire growth season and for germination range in between 20 and 22 °C and 25 to28°C respectively, whereas during the day and at night the optimum temperature is 25 to 33 °C and 17 to 23 °C correspondingly^64^. Notably, excessive temperatures exceeding 30 °C from June to August reduced maize yield. Moreover, in the pre-flowering and post-flowering stages of the maize life cycle, temperatures between 33 and 36 °C decrease the CO_2_ exchange rate (~ 17%), crop growth rate (17–29%), grain number (7–45%), and grain yield (10–45%)^65^. For maximum growth, maize needs 500 to 900 mm of rainfall during the growing season^66^whereas wheat requires 400 to 1100 mm^67^. Prior to and throughout maturity, severe rains can cause lodging, losses of yield, and quality degradation of wheat and therefore, the volume and distribution of rainfall during the season had a greater impact on yield^68]– [69^. These studies clearly demonstrate how these two phenomena affect wheat and maize yields, highlighting the urgent need for future research aimed at minimizing climate impacts through sustainable agronomic approaches and the development of climate-smart agricultural practices. Adjusting sowing times may serve as an effective strategy to mitigate the adverse effects of climate change on wheat and maize yield^52,70^. As climate change continues to intensify the frequency and severity of heatwaves and droughts, understanding their effects on crop production processes and development of adaptation management strategies are essential for improving resilience in maize and wheat production systems in the country.

Accurate crop yield prediction is crucial for resource use optimization, crop management practices, food security and effective decision making in agricultural systems^71–73^. A crop modeling study aimed at predicting the impact of climate change on crop production in Bangladesh is essential to understanding the country’s future agricultural challenges. There are different modeling approaches are being used for wheat and maize crops yield prediction such as process based crop growth models^18,74–84^. Although these crop models are widely used and offering insights to assess climate change and climate variability impacts on wheat and maize crops in different regions under contrasting climates^16,85–89^. These models use mechanistic representations of physiological and growth processes and require detailed inputs, such as weather, soil properties, and crop management data. These studies use extensive and quality data by adopting crop, and climate models to simulate how changing climatic factors (temperature, rainfall, and CO2 concentrations) will influence crop yields in different regions^90–96^. A study using a Crop Simulation Model Inter Comparison Approach by Chawdhery et al.,^97^ in Bangladesh recommended two major strategies for increasing the yield and enhancing profit in a rice-wheat cropping system—shifting showing time for wheat and increasing irrigation volume. Similarly, a study by Teixeira et al.,^98^ suggests that wheat and maize yields may also be severely impacted due to increased heat stress and altered monsoon dynamics. However, these models’ performance depends on high quality extensive input data for calibration and evaluation before application in climate change impact assessment and adaptation strategies development for sustainable crops production^2,96,99–104^. In contrast, machine learning (ML) modeling approaches especially Fuzzy logic models have emerged as powerful tools for yield prediction due to its ability to handle imprecise, uncertain, and nonlinear relationship between yield and wide range of input variables^72,105–108^. Fuzzy logic models are recently being applied for crop yield prediction and for climate change impact assessment. By providing projections under different climate scenarios, Fuzzy logic model helps inform adaptive measures, such as the adoption of climate-resilient crop varieties, improved water management, and shifts in planting seasons, crucial for safeguarding Bangladesh’s food security in the face of climate change^99,109–111^. Fuzzy logic deals with partial truth^112^and encompasses methods like Mamdani, Sugeno, and Tsukamoto^113^. Fuzzy logic’s ability to handle uncertain, imprecise, and vague data makes it valuable in various fields such as agriculture, biomedicine, and earth science, offering significant opportunities for research and development. A fuzzy inference system involves fuzzification, inference, and defuzzification, where crisp quantities are converted to fuzzy and vice versa^114^. This research applied one of the Fuzzy logic model approaches viz. the Mamdani method to predict the impact of climate change on winter wheat, winter maize, and summer maize yield.

To the author’s best knowledge, no modelling study has been conducted in the study area (Rajshahi, Bangladesh) to predict the impact of climate change on crop yield, particularly by fuzzy logic modeling approach based on different phenological stages (Table 1). The specific objectives for this study are (i) to create a fuzzy logic model that incorporates key climate variables (temperature, precipitation) and their non-linear effects on wheat and maize (winter and summer) yield including a comparison with global yield scale (ii) to identify critical climatic stress factors (elevated temperature and precipitation) that most significantly affect wheat and maize yields, and how these will be represented in the fuzzy logic system and (iii) to reconsider suitable sowing time based on fuzzy prediction and long-term climate data for minimizing climate change impact on wheat and maize (winter and summer) yield in the study region.

**Table 1:** Available literature of climate variability on wheat and maize with application of fuzzy logic modelling approach.

Country	Title	Key findings	References
Bangladesh	Assessing the impacts of climate change on cereal production in Bangladesh: evidence from ARDL modeling approach	The study found that rainfall has a positive impact on cereal production in both the short and long-term.	(Chandio et al., 2022)
Bangladesh	Impact Study of Climatic Variability on the Productivity of Major Crops in South Western Part of Bangladesh Using Fuzzy Logic	Climate change will negatively impact crop productivity in Bangladesh. Simulation study using historic climate data can help determine policy options and research needs.	(Shahadat et al., 2021)
India	Fuzzy Expert system for the Impact of Climate Change in Indian Agriculture	There are numerous negative effects on agriculture resulting from unfavorable changes in the environment.	(Karthika et al., 2018)
China	Impacts of climate change on drought risk of winter wheat in the North China Plain	Temperature increases will result in more frequent droughts and lower winter wheat yields.	(Zhang et al., 2021)
Germany	Site-specific impacts of climate change on wheat production across regions of Germany using different CO2 response functions	Climate change will have varying impacts on wheat production across Germany. Different CO2 response functions affect yield, groundwater recharge, and nitrogen leaching.	(Kersebaum and Nendel, 2013)
France	A meta-analysis of the predicted effects of climate change on wheat yields using simulation studies	Wheat yields are likely to increase with higher CO2 concentrations and moderate declines in precipitation. Results vary depending on local soils, farming practices, and other factors.	(Wilcox and Makowski, 2014)
Australia	Improving productivity of Australian wheat by adapting sowing date and genotype phenology to future climate	Similar to most cereals, wheat can be negatively impacted by heat and water stress during pollination, which also affects grain size after the reproductive phases.	(Collins and Chenu, 2021)
South Africa	Yield reduction under climate warming varies among wheat cultivars in South Africa	It is expected that South Africa’s irrigation demand will rise 6.4% annually through 2050 as a result of the expected dry weather, further emphasizing the country’s restricted water supply.	(Shew et al., 2020)
Ghana	Effect of rainfall and temperature variability on maize yield in the Asante Akim North District, Ghana	Rising yearly rainfall and temperature patterns positively impact the yield of maize	(Baffour-Ata et al., 2023)
USA	Projections of spring wheat growth in Alaska: Opportunity and adaptations in a changing climate	Minimum temperature, precipitation, and precipitation scarcity are the climate variables that traditionally restrict springtime wheat yield.	(Harvey et al., 2021)
India	Rain Prediction using Fuzzy Logic	Fuzzy logic is used in situations when exact inputs are not required and it can function without a mathematical model that maps inputs to outputs.	(Singla et al., 2019)
Indonesia	Study of a Weather Prediction System Based on Fuzzy Logic Using Mamdani and Sugeno Methods	In an unpredictable environment, fuzzy systems can create intelligent systems.	(Setyanugraha et al., 2022)

## Materials and methods

### Study area

**Fig. 1:**
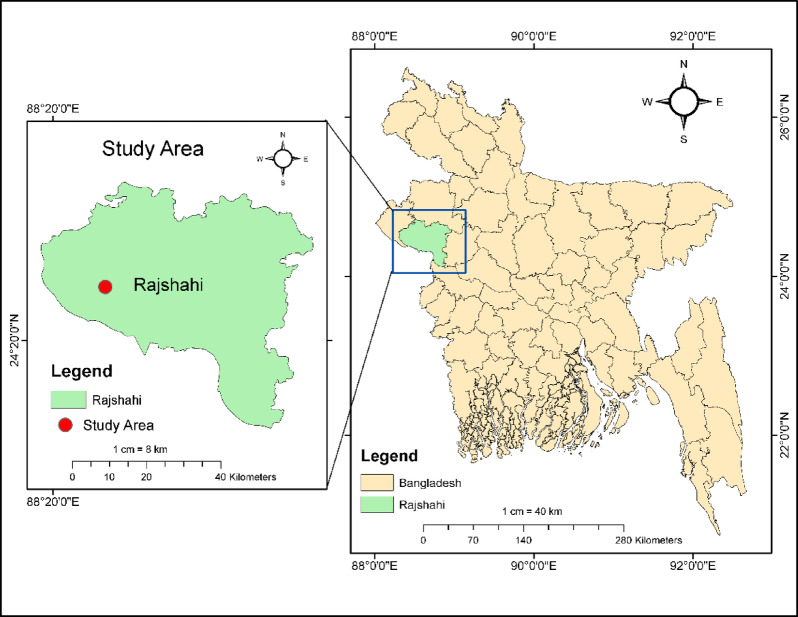
The study area: Rajshahi, Bangladesh (drought-prone agroecological zone (AEZ) of Northwestern Bangladesh).

In Bangladesh’s north-west regions, drought occurs frequently^115^. For this study, Rajshahi district was purposefully selected from the drought-prone agroecological zone (AEZ) of northwestern Bangladesh (Fig. 1). The area around Rajshahi town (only 22 m a.sl.) is one of the largest wheat and maize cultivating regions in north-western Bangladesh and has a (sub-) tropical monsoon climate with a distinct cool (winter) dry season from November to March, and a hot to warm (summer) wet season from April to October. The district experiences an annual mean temperature of 25.5 °C. The amount of precipitation that Rajshahi receives annually is 1466 mm^116^. Most (> 80%) of the precipitation in Rajshahi falls between July and early September. In Rajshahi district, the growing seasons are as follows: wheat from November to March, maize winter from November to April, and maize summer from April to July.

### Data collection

The research team collected annual yield data (2001 to 2023) for winter wheat and both winter and summer maize in Rajshahi from the Department of Agricultural Extensions (DAE), Rajshahi; and Bangladesh Bureau of Statistics (BBS). The research team collected global wheat and maize yield data, along with information on the top producing countries data, from FAOSTAT. Temperature and rainfall data of Rajshahi, covering the period from 2000 to 2024 were obtained from the Bangladesh Meteorological Department (BMD), in A summary of the data collection is provided below:

**Table:** 

Category	Parameter	Number of samples	Collection period	Data sources	Methods of collection
Crop Yield	Wheat and maize yield (winter and summer seasons)	23 (yearly data)	2001–2023	BBS; DAE, Rajshahi	Official reports and records
	Global yield data	23 (yearly data)	2001–2023	FAOstat	Official reports and records
Climate Data	Temperature	25 (yearly data)	2000–2024	BMD	Purchased data
	Rainfall	25 (yearly data)	2000–2024	BMD	Purchased data

### Fuzzy logic modeling approach

**Fig. 2:**
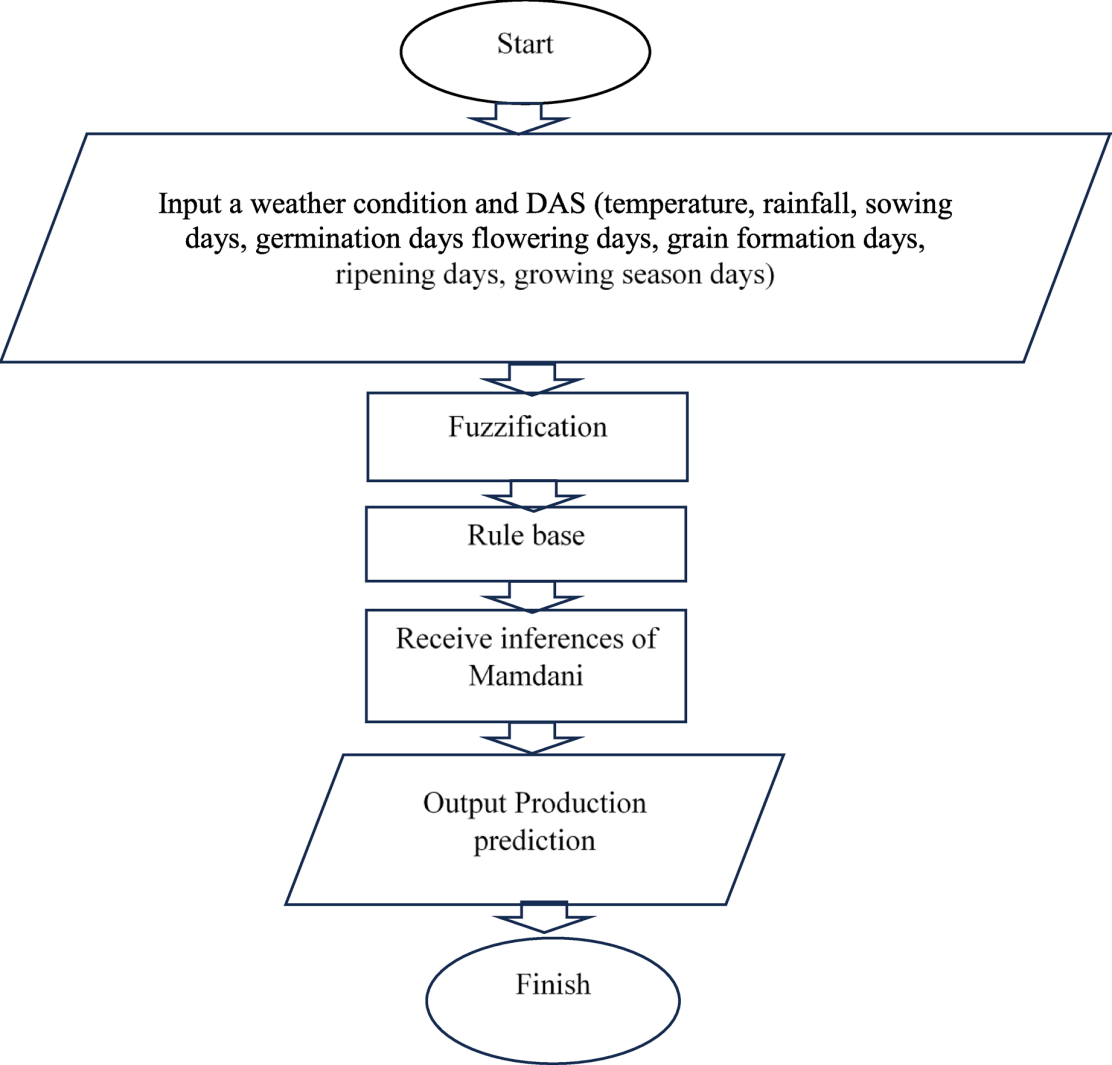
Yield prediction flowchart using Fuzzy modeling approach.

**Fig. 3:**
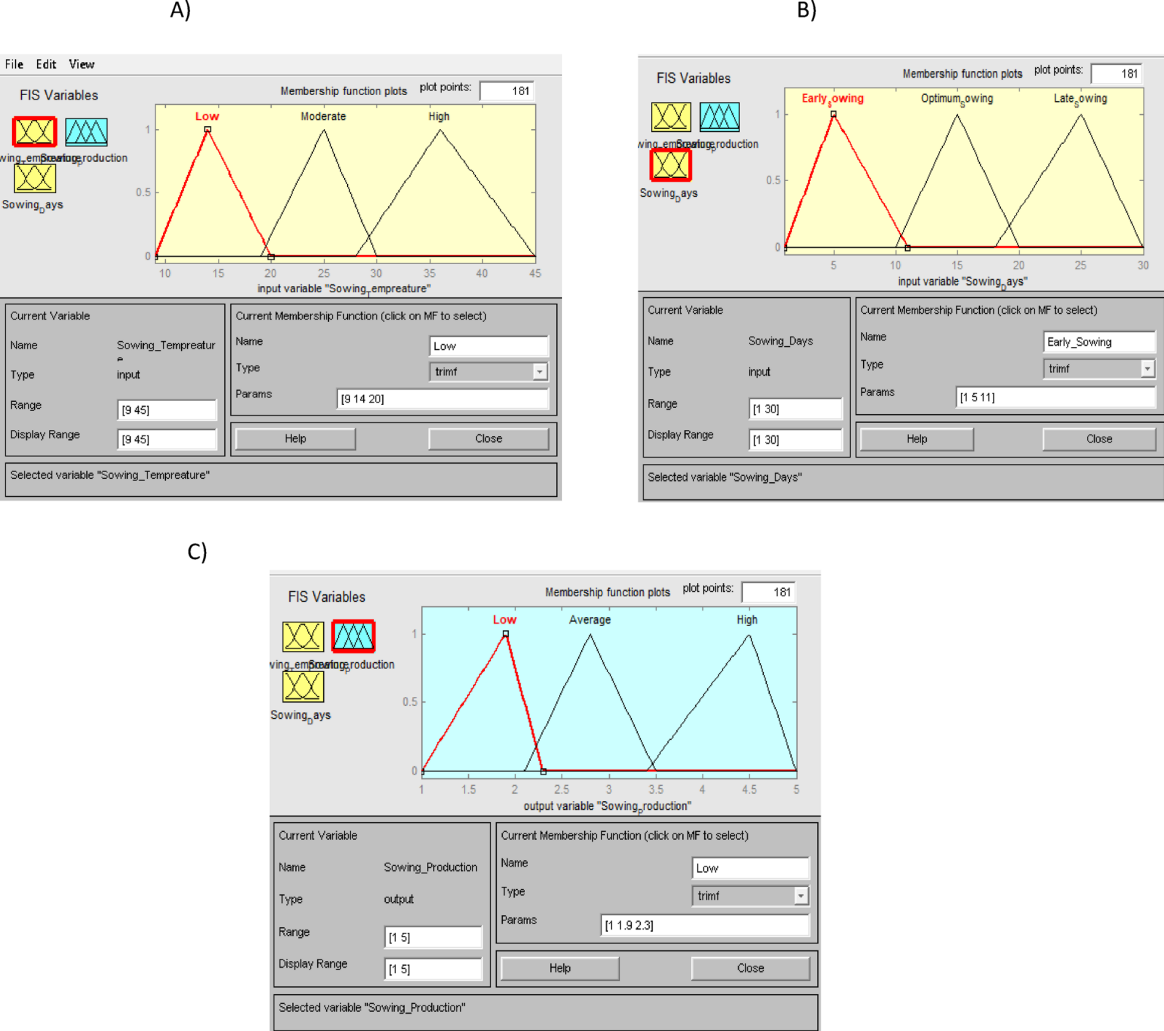
Linguistic variables and memberships function for sowing time in wheat yield; (**A**) represents linguistic variables and membership’s function for Temperature, (**B**) represents for Days and (**C**) represents for Production (Yield).

In fuzzy logic, linguistic variables utilize words or sentences from natural or artificial language as their values. ​These variables are very common in fuzzy models. For example, the air temperature (°C) could be defined as a linguistic variable if its values are linguistic rather than numerical, i.e., cold, warm, hot, rather than 9 °C, 22 °C and 38 °C. As in this research, the Mamdani method was employed to predict the yield of wheat crop, maize winter and maize summer . This modeling approach consisted of four primary steps to produce an output value, fuzzification, implication of function (rule) application, combination of rules (aggregation) and defuzzification, respectively. The schematic diagram illustrating the yield prediction flowchart based on fuzzy modeling is presented in Fig. 2. At the first step, input value was converted into fuzzy as linguistic value. Temperature, DAS, rainfall are the input values and yield are the output value for wheat crop, and both winter and summer maize. For each crop, six growth stages were considered (whole growing season, sowing time, germination, flowering, grain filling, and ripening, respectively) and analyzed using the Mamdani method to predict crop yields effectively. Figure 3. shows the membership function of the fuzzy input temperature, rainfall, DAS and output yield for wheat growing season as well as for sowing time. In input section temperature [9–45] °C has the linguistic form of low [9 14 20] °C, moderate [19 25 30] °C, high [28 36 45] °C; Rainfall [0-1600]mm has light [0 250 500]mm, moderate [450 700 1000]mm, heavy [950 1200 1600] mm as linguistic variables and early sowing [1 5 11] days, optimum sowing [10 15 20] days, late sowing [18 25 30] days, respectively are the linguistic form of DAS [1–30] days. And for yield, the membership function has the linguistic form as low [1 1.9 2.3] t ha^− 1^, average [2.1 2.8 3.5] t ha^− 1^ and high [3.4 4.5 5] t ha^− 1^ for wheat. In case of winter and summer maize yield, linguistic variables and membership functions are shown in Table S1**.**

For the second step, we used triangular membership function formula as the input and output values have the triangular form. In fuzzy logic, the formula for triangular membership function is defined as,

$$\:\mu\:\left(x\right)=\left\{\begin{array}{c}0\:\:\:\:\:\:\:\:\:\:\:\:\:\:\:if\:x\le\:a\\\:\frac{x-a}{b-a}\:\:\:\:if\:a<x\le\:b\\\:\frac{c-x}{c-b}\:\:\:\:\:if\:b<x\le\:c\\\:0\:\:\:\:\:\:\:\:\:\:\:\:\:\:\:if\:x>c\end{array}\right.$$

Where a, b and c are the parameters of the triangle. By using this mathematical formula, we find the following membership functions for growing wheat and sowing stages input and output.

Now, we are showing only one input variable’s membership functions calculation process. For the rest of the input and output, we followed the same mathematical formula and process.

For temperature [9–45] $$\:℃$$

$$\:{\mu\:}_{Low}\left(x\right)=\left\{\begin{array}{c}0;\:\:\:\:\:x\le\:9\\\:\frac{x-9}{14-9}=\frac{x-9}{5};\:\:\:9<x\le\:14\\\:\frac{20-x}{20-14}=\frac{20-x}{6};\:\:14<x\le\:20\\\:0;\:\:\:\:\:x\ge\:20\end{array}\right.$$

$$\:{\mu\:}_{Moderate}\left(x\right)=\left\{\begin{array}{c}0;\:\:\:\:\:x\le\:19\\\:\frac{x-19}{25-19}=\frac{x-19}{6};\:\:\:19<x\le\:25\\\:\frac{30-x}{30-25}=\frac{30-x}{5};\:\:25<x\le\:30\\\:0;\:\:\:\:\:x\ge\:30\end{array}\right.$$

$$\:{\mu\:}_{High}\left(x\right)=\left\{\begin{array}{c}0;\:\:\:\:\:x\le\:28\\\:\frac{x-28}{36-28}=\frac{x-28}{8};\:\:\:28<x\le\:36\\\:\frac{45-x}{45-36}=\frac{45-x}{9};\:\:36<x\le\:45\\\:0;\:\:\:\:\:x\ge\:45\end{array}\right.$$.

By following the same process, we obtained all of the triangular membership function for various input range including for winter and summer maize.

For the third step, combination of rules, we used IF-THEN rule which are shown in the (Table 2). Finally, for finding the output from fuzzy value to crisp or classical value we used the defuzzification process. Several approaches or techniques can be used for the defuzzification process. The used method which is known as centroid method (Centre of Gravity). The defuzzification value $\:{Z}^{*}$ using center of gravity is defined as,

**Table 2:** Fuzzy IF-THEN modelling rules for wheat and maize yield prediction.

	Wheat (winter)			Maize Winter			Maize Summer		
**Stages**	**Temp.**	**Days**	**Yield**	**Temp.**	**Days**	**Yield**	**Temp.**	**Days**	**Yield**
Sowing Time	Low	Early	Low	Low	Early	Low	Low	Early	Low
		Optimum	Average		Optimum	Low		Optimum	Low
		Late	Low		Late	Low		Late	Low
	Moderate	Early	Average	Moderate	Early	High	Moderate	Early	Average
		Optimum	High		Optimum	High		Optimum	High
		Late	Average		Late	Average		Late	Average
	High	Early	Low	High	Early	Average	High	Early	Average
		Optimum	Average		Optimum	Average		Optimum	Low
		Late	Low		Late	Low		Late	Low
Germination and Early Growth Stages	Low	Early	Low	Low	Early	Low	Low	Early	Low
		Optimum	Average		Optimum	Average		Optimum	Low
		Late	Low		Late	Average		Late	Low
	Moderate	Early	Average	Moderate	Early	Average	Moderate	Early	High
		Optimum	High		Optimum	High		Optimum	High
		Late	Average		Late	High		Late	Average
	High	Early	Low	High	Early	Average	High	Early	Average
		Optimum	Average		Optimum	Average		Optimum	Average
		Late	Low		Late	Low		Late	Low
Flowering Stage	Low	Early	Average	Low	Early	High	Low	Early	Low
		Optimum	Average		Optimum	High		Optimum	Low
		Late	Low		Late	Average		Late	Low
	Moderate	Early	Average	Moderate	Early	Average	Moderate	Early	Average
		Optimum	High		Optimum	Average		Optimum	Average
		Late	Average		Late	Average		Late	Average
	High	Early	Average	High	Early	Average	High	Early	High
		Optimum	Low		Optimum	Low		Optimum	High
		Late	Low		Late	Low		Late	Average
Grain Formation Stage	Low	Early	Average	Low	Early	High	Low	Early	Low
		Optimum	Average		Optimum	High		Optimum	Low
		Late	Low		Late	Average		Late	Low
	Moderate	Early	High	Moderate	Early	Average	Moderate	Early	High
		Optimum	High		Optimum	Average		Optimum	High
		Late	Average		Late	Average		Late	Average
	High	Early	Low	High	Early	Average	High	Early	Average
		Optimum	Low		Optimum	Low		Optimum	Low
		Late	Low		Late	Low		Late	Low
Ripening Stage	Low	Early	Average	Low	Early	Low	Low	Early	Low
		Optimum	High		Optimum	Low		Optimum	Low
		Late	Average		Late	Low		Late	Low
	Moderate	Early	Average	Moderate	Early	High	Moderate	Early	Average
		Optimum	Average		Optimum	High		Optimum	Average
		Late	Low		Late	Average		Late	Average
	High	Early	Low	High	Early	Average	High	Early	High
		Optimum	Low		Optimum	Low		Optimum	High
		Late	Low		Late	Low		Late	Average
Whole growing Season	**Temperature**	**Rainfall**	**yield**	**Temperature**	**Rainfall**	**yield**	**Temperature**	**Rainfall**	**Yield**
	Low	Light	Low	Low	Light	Low	Low	Light	Low
		Moderate	Average		Moderate	Average		Moderate	Average
		Heavy	Low		Heavy	Average		Heavy	Average
	Moderate	Light	Average	Moderate	Light	Average	Moderate	Light	Low
		Moderate	High		Moderate	High		Moderate	High
		Heavy	Average		Heavy	High		Heavy	High
	High	Light	Low	High	Light	Average	High	Light	Low
		Moderate	Low		Moderate	Average		Moderate	High
		Heavy	Low		Heavy	Low		Heavy	High

$$\:{Z}^{*}=\frac{\sum\:_{i=1}^{N}{A}_{i}\times\:{\overline{Z}}_{i}}{\sum\:_{i=1}^{N}{A}_{i}}$$

Here, the variable N denotes the total number of sub-areas, whereas the variables $\:{A}_{i}$ and $\:{\overline{Z}}_{i}$ represent for the area and the centroid, respectively, of the $\:{i}^{th}$sub-area.

### Tools and software

The software R was used for descriptive analysis (confidence level of 95%) and the correlation test to compare wheat and maize yield among Rajshahi, Bangladesh and world. The MATLAB (7.0) software and fuzzy toolbox for fuzzy logic were used to estimate the wheat and maize yield in different climatic scenarios (elevated temperature and rainfall variability) and further to predict the climate impact on wheat and maize yield in the future. Furthermore, sowing time adaptation for wheat and maize crop (winter and summer seasons) was also predicted for sustainable yield prediction in future.

## Result

### Descriptive analysis of wheat and maize crop yield

The wheat yield in Rajshahi, Bangladesh, and globally ranged from 1.59 t ha^− 1^ to 3.89 t ha^− 1^, 1.53 t ha^− 1^ to 3.69 t ha^− 1^, and 2.65 t ha^− 1^ to 3.70 t ha^− 1^, respectively (**Table S2**). In Rajshahi, the highest wheat yield recorded in 2021 (3.89 t ha^− 1^), while the lowest 1.59 t ha^− 1^, was observed in 2005. In addition, wheat yield in Bangladesh peaked at 3.69 t ha^− 1^ in 2023, a significant increase from the minimum of 1.53 t ha^− 1^ in 2006. However, global wheat yield reached 3.70 t ha^− 1^ in the year 2022.

The winter maize yields in Rajshahi ranged from 3.17 t ha^− 1^ to 10.48 t ha^− 1^, whereas summer maize yields fluctuated between 2.26 t ha^− 1^ and 7.48 t ha^− 1^ (Table S3). The highest yield of winter maize was produced in 2021 (10.48 t ha-1) whereas lowest recorded in 2001 (3.17 t ha^− 1^). In contrast, summer maize yield was maximum recorded in 2020 accounted by 7.48 and minimum was obtained from the same year as winter maize (**Table S3**). In Bangladesh, maize yield varied from 2.06 to 9.25 t ha^− 1^, while global yield falls between 4.32 and 5.96 t ha^− 1^. In both contexts, maize yields had shown a gradual increase from the year 2000 to 2023. In 2000, the minimum maize yield in Bangladesh and globally was recorded at 2.06 t ha^− 1^ and 4.32 t ha^− 1^, respectively. Conversely, the maximum maize yield figures were 9.25 t ha^− 1^ for Bangladesh and 5.96 t ha^− 1^ for the globe

#### Comparative analysis of wheat and maize yields in rajshahi, bangladesh, and global

**Fig. 4:**
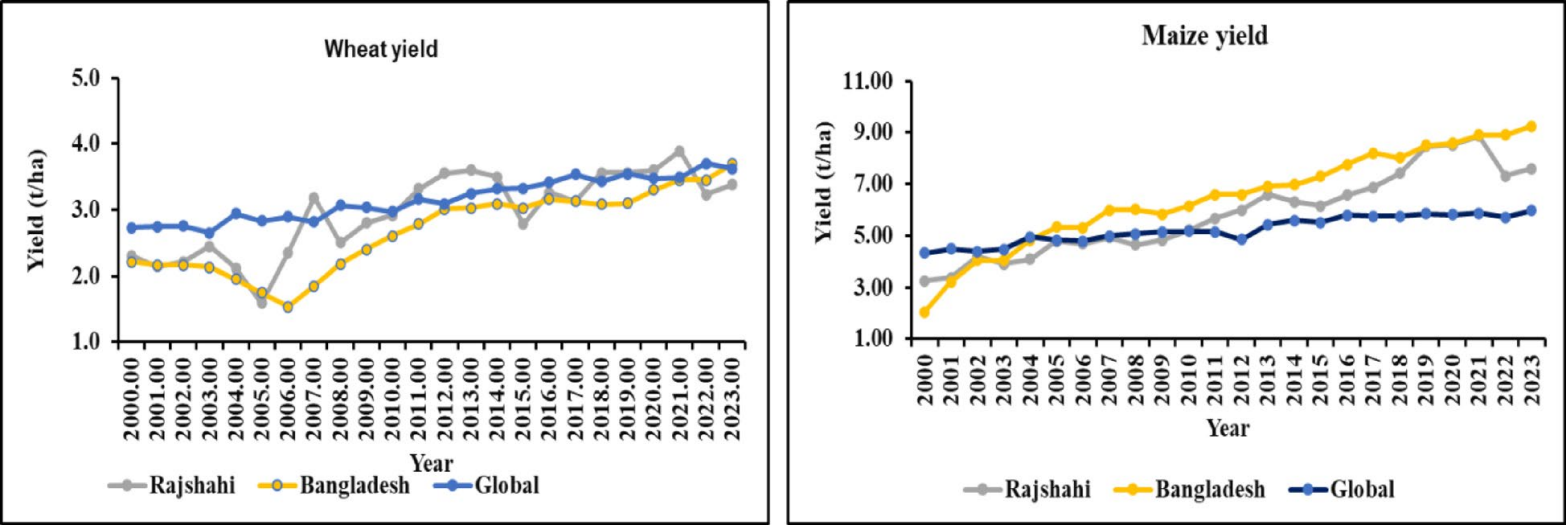
Wheat and maize yield comparison among Rajshahi, Bangladesh, and global.

Regarding wheat crop, Rajshahi’s yield fell short of the global average until in 2007 and varied significantly between 2000 and 2009. Between 2010 and 2014, Rajshahi’s output rate outpaced both Bangladesh’s and the global average (Fig. 4). Wheat yield varied between 2015 and 2018, then again overtook Bangladesh and the global until 2021. In 2022 and 2023, the Rajshahi yield rate dropped significantly (Fig. 4). On the other hand, the yield of maize in Rajshahi was above the global yield but below Bangladesh (Fig. 4). Although the yield rate in Rajshahi fluctuated in several periods, Bangladesh showed a gradual increase from 2000 to 2023. However, the global scale showed an almost constant rate of production in those years (Fig. 4).

#### Comparison of wheat and maize yield with top-growing countries

**Fig. 5:**
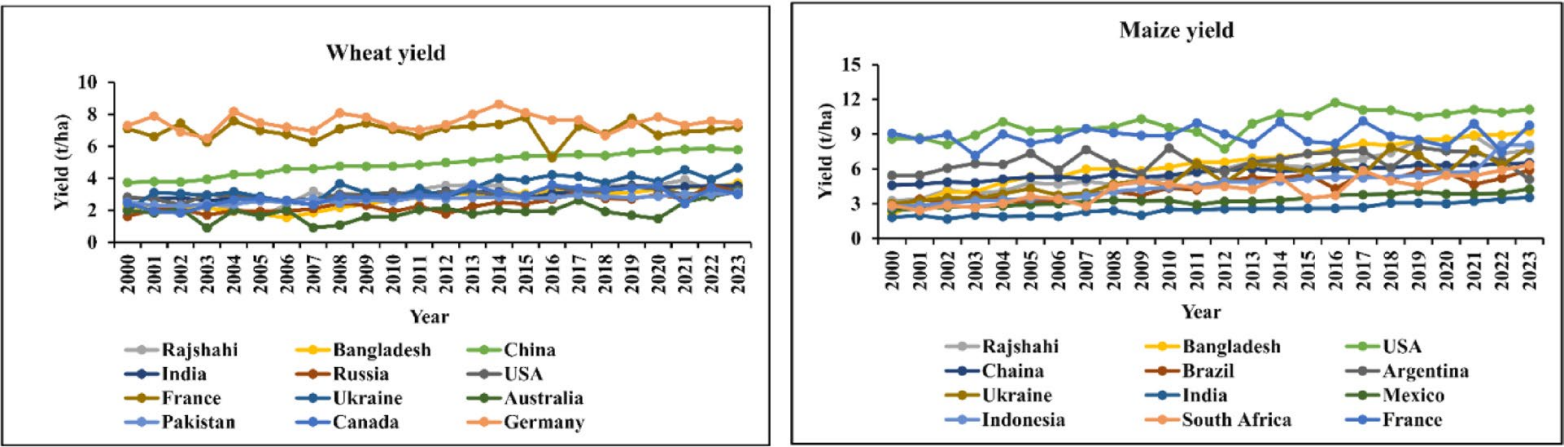
Comparing the wheat and maize yield of Rajshahi and Bangladesh to the global top-growing countries (2001–2023).

When it comes to wheat yield, Germany leads with constant variation, except for 2002 and 2019, whereas Rajshahi showed below-average results (Fig. 5). Australia is near the graph’s bottom line, while France exhibits significant volatility in 2016, while China has a stable yield position (Table S4). However, the USA has been the top maize-growing country for many years though the yield rate decreased substantially during 2012 (Fig. 5). When compared to other leading maize-growing nations, Bangladesh’s yield rate is likewise excellent, while Rajshahi’s output is nearly as high as Bangladesh’s (Fig. 5). In contrast to those nations that exhibit consistent yield, such as India, Mexico, Indonesia; France, Ukraine, and South Africa displayed significant fluctuations (Table S5).

### Correlation analysis of wheat and maize yield with Bangladesh and global

**Fig. 6:**
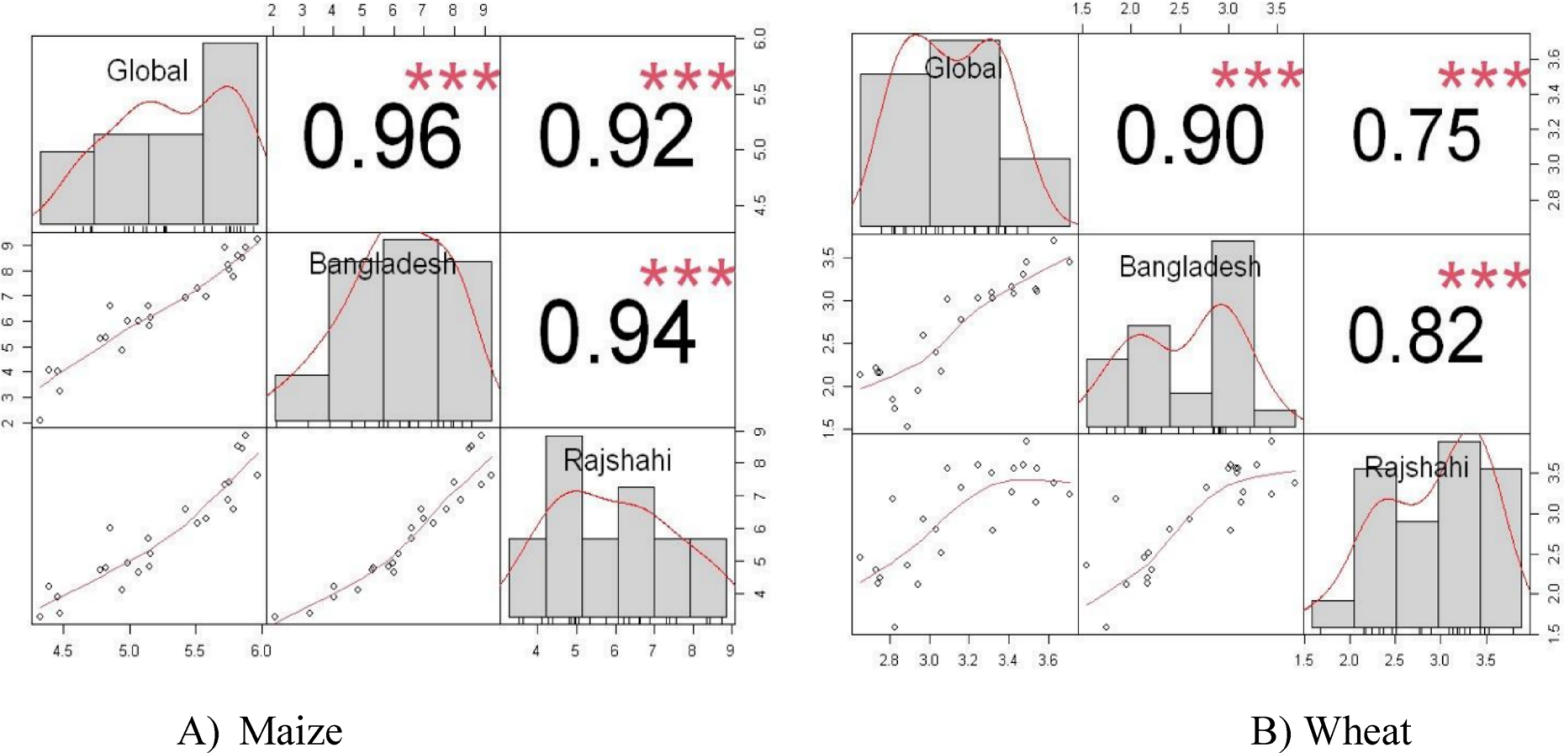
Correlation of wheat and maize yield of Rajshahi with Bangladesh and global yield. (**A**) represents maize correlation and (**B**) represents wheat correlation. (* indicates significance level).

We explored relationship between yield of wheat in Rajshahi with Bangladesh and global scale (Fig. 6B). The global wheat yield shown a highly significant and strong positive relationship with Bangladesh (0.90) and Rajshahi (0.75). Moreover, Rajshahi demonstrated a highly significant and positive relation with wheat yield of Bangladesh (0.82). On the other hand, as like wheat, yield of maize at Rajshahi had a highly significant and strong positive relationship with Bangladesh (0.94) and global scale (0.92). Similarly, the maize yield in Bangladesh had also a highly significant and positive correlation with global maize yield (0.96) (Fig. 6A).

### Correlation analysis of wheat and maize yield with rainfall and temperature

**Fig. 7:**
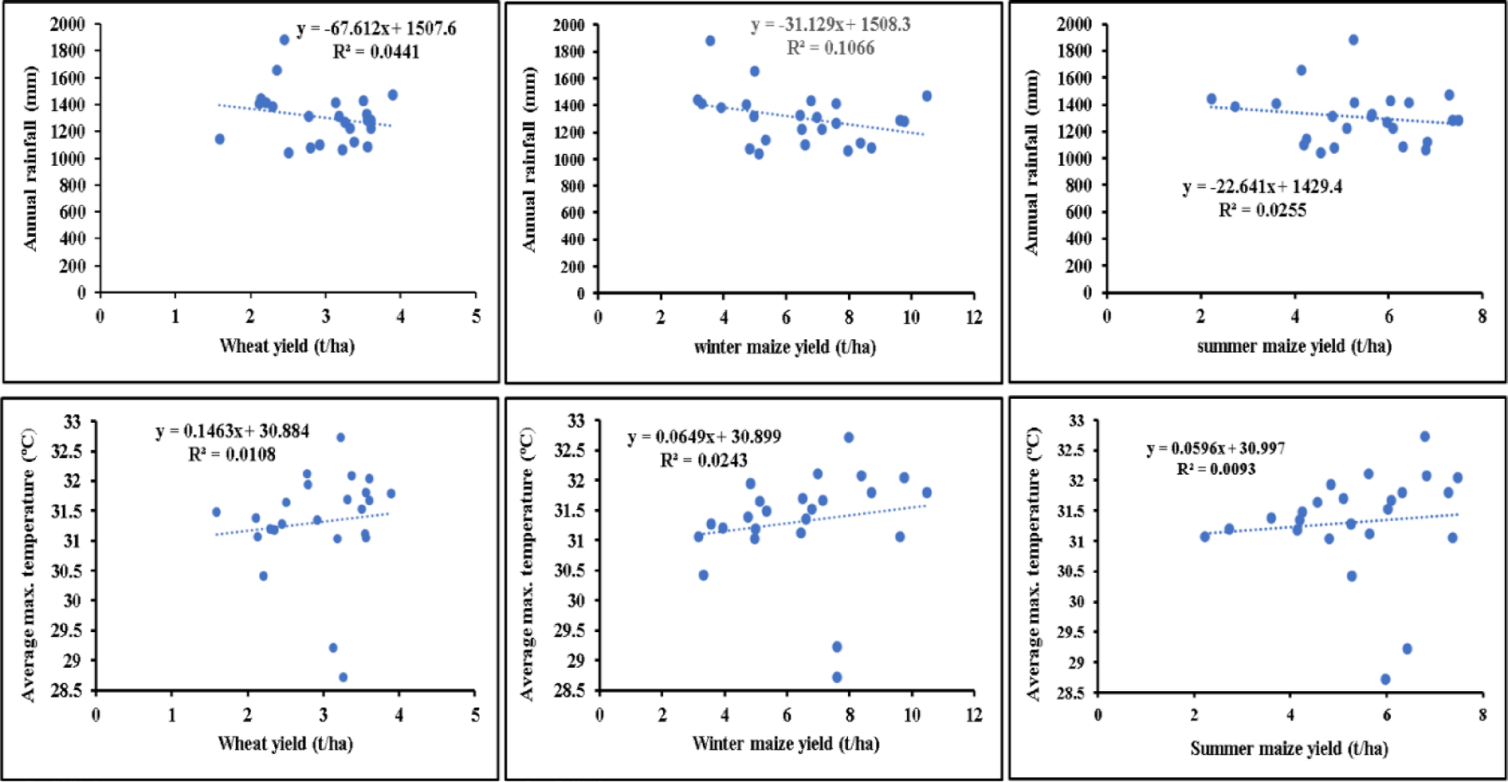
Correlation analysis of winter wheat and maize (winter and summer) yield with temperature and rainfall. Dotted line refers to the regression line and blue-colored circle represents the annual rainfall and average maximum temperature. R^2^ is the square of the correlation coefficient (r).

With a correlation coefficient of -0.2099 (R^2^ = 0.0441), a weak negative correlation is indicated between wheat yield and annual rainfall (Fig. 7). This implies that, although the association is weak, wheat yield tends to decline slightly as annual rainfall rises. On the other hand, a very modest positive correlation is indicated (R^2^ = 0.0108) for wheat and average maximum temperature (Fig. 7). This indicated that the link between average maximum temperature and wheat yield is essentially nonexistent.

There is a weak negative link, as indicated by the correlation coefficient of -0.1598(R^2^ = 0.0255) is found between annual rainfall and summer maize yield (Fig. 7). This implies that lower summer maize yields are marginally correlated with higher year rainfall. However, given R^2^ = 0.0093, a very modest positive correlation is indicated between summer maize yield and average maximum temperature. This implies that the yield of summer maize and average maximum temperature have virtually no correlation. However, a moderately negative correlation is shown by the R^2^ =0.1066 and shows that lower winter maize yields are linked to higher annual rainfall. In contrast, average maximum temperature shows a weak positive link with winter maize yield (R^2^=0.0243) (Fig. 7). According to this, there may be a small but weak correlation between higher average maximum temperatures and higher winter maize yields.

### Climate change impact scenarios in Rajshahi

#### Yield of winter wheat and maize in relation to rainfall (2001–2023)

**Fig. 8:**
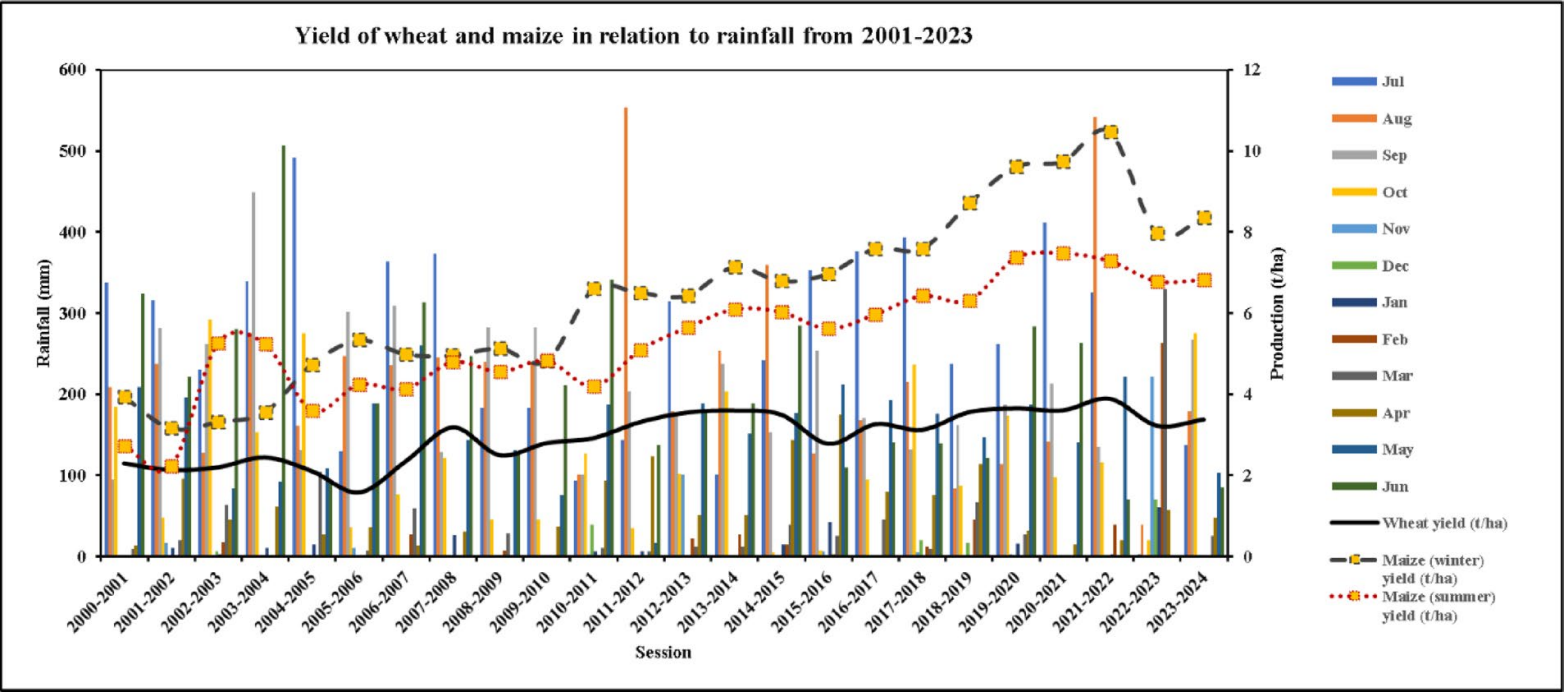
Yield of winter wheat and maize (winter and summer) in relation with rainfall (2001–2023) in Rajshahi, Bangladesh.

The relation between the yield of winter wheat and rainfall has shown in Fig. 8, where how the yield was affected by the minimum rainfall during each phenological stages (November-April) is demonstrated. The yield of the wheat increased gradually although the amount of rainfall fluctuated throughout the years in every season (Table S6). In 2012–2013 wheat yield increased as compared to that of previous year as the suitable rainfall occurred in the month of November (101 mm) (Fig. 8). Almost every growing year recorded a modest increase in yield, with the exception of 2015–2016, when there was less rainfall during the wheat growing season than in previous years; however, from 2020 to 2024, there is virtually no rainfall during the growing season, resulting in a lower yield than in previous years (Fig. 8).

The growing season of winter maize begins from November-February whereas summer maize April to July. From Fig. 8, it is recognized that there is evidence of the effect of rainfall on winter wheat. The figure also shows the relationship between the winter and summer maize yield with the rainfall variability. The yield of winter maize was comparatively higher than the summer maize due to existing favorable temperature and rainfall during the pre-growing season (Fig. 8). During the growing season of winter maize (November-February), no significant rainfall was occurred, but the yield was about to increase with little decrease as almost 78% lands are under irrigated condition in study area^117]– [118^. However, summer maize yield can’t reach up to the mark though it gets enough rainfall due to the high temperature over the growing season. This finding is harmony with the findings of Asseng et al.,2015 ^122^ stated excessive temperature above 30 °C significantly caused reduction of corn yield.

#### Yield of winter wheat and maize in relation to temperature (2001–2023)

**Fig. 9:**
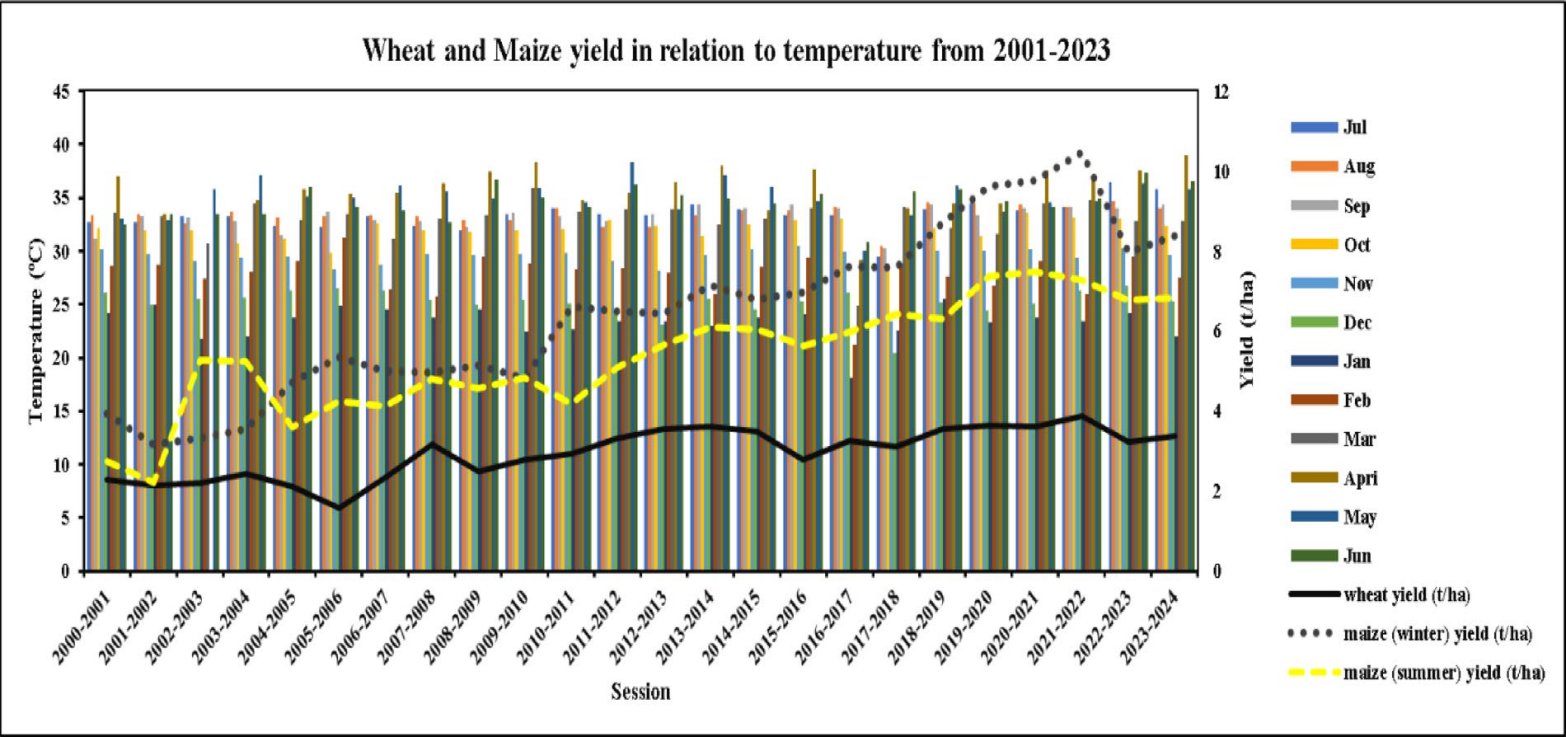
Yield of winter wheat and maize (summer and winter) in Rajshahi with relation to temperature (2001–2023) in Rajshahi, Bangladesh.

It was found that the temperature had a less influence on winter wheat yield as the temperature is low during the growing season. Figure 9 showed the temperature did not vary too much over the years as well as the yield. The yield rate decreased during 2005–2006, 2008–2009, 2015–2016 and 2022–2023 as the average maximum temperature of that growing year was high compared to others (**Table S7**). However, without some exceptional years, there were minor effects of temperature on winter wheat are found.

A significant effect of temperature on maize yield was observed in Rajshahi from 2001 to 2023 as maize is sensitive to heat stress as shown in Fig. 9. By analyzing the figure, in general, the yield of both winter and summer maize were increased over the times. Noticeably, winter maize yield was always higher than the summer maize except 2002–2004, due to the favorable temperature prevailed during the growing season of winter maize (average 16–20 °C) whereas, high temperature reduced the summer maize yield. Because, in summer season, day temperature exceeds 30 °C in the study area (**Table S7**). This finding is similiar with the result of Zhang et al. (2021)^120^ reported that the yield of maize dropped by 1–1.7% for every degree Celsius increase (over 30 °C). However, after 2021–2022, both the winter and summer maize especially winter maize yield declined suddenly with the rising of the average maximum temperature (**Table S7**). Then, the yield commenced to rise again. The highest yield of winter maize (10.48) was noticed in the year 2021-22 since the growing season temperature was favorable than other years.

### Climate change impact analysis through fuzzy logic modelling approach

Various logic (IF-THEN rules) for individual phenological stage of wheat and maize have been developed and presented in Table 2. The numerous phases of phenological growth (sowing, germination, flowering, grain filling, and ripening) of both wheat and maize crops have a notable effect on the ultimate grain yield of these crops. In this experiment, we used the fuzzy logic system to determine the effect of climate variability on different growth stages of wheat and maize and its ultimate impact on yield. The optimum sowing time is crucial to get successful crop yield. For both wheat and maize, there is optimum sowing time and temperatures ranges. Consequently, during this period, fuzzy shows the maximum output. However, beyond this limit, the yield declines substantially. The research team finds that in fuzzy system,  the maximum wheat yield will be 4.28 tons/ha at 27 °C and 15 DAS (days after sowing), whereas at 30 °C and 10 DAS, the yield will decline to 1.67 tons ha^− 1^ (Table 3).

**Table 3:** Modelling the effect of temperature and rainfall variability on wheat and maize yield using fuzzy logic modelling Approach.

	Wheat			Maize Winter			Maize Summer		
Sowing Time	Temp. (°C)	Days	Yield (t ha^− 1^)	Temp. (°C)	Days	Yield (t ha^− 1^)	Temp. (°C)	Days	Yield (t ha^− 1^)
	27	15	4.28	27	25	9.57	28	5	5.8
	22	12	4.25	20	40	7	35	17	4
	20	13	4.22	35	35	4.5	32	23	4
	30	10	1.67	24	30	7.16	22	12	7.74
	25	10	2.8	29	27	8.66	19	26	4
Germination and Early Growth Stages	28	32	2.8	28	33	7.16	24	13	7.7
	19	12	1.67	30	30	7.16	30	23	5.8
	25	20	4.28	24	44	9.57	33	30	4
	30	25	2.8	26	45	9.59	22	35	5.8
	22	30	2.8	20	33	7.16	42	26	5.8
Flowering Stage	27	105	4.28	20	95	7.16	25	63	5.8
	10	100	2.8	10	100	9.59	32	78	7.74
	20	105	4.22	17	110	7.16	37	87	5.8
	30	110	1.68	25	115	7.17	40	72	7.74
	25	97	4.28	15	103	9.57	34	69	7.75
Grain formation Stage	23	115	4.28	19	110	9.59	25	83	7.72
	27	110	3	23	120	7.16	29	97	6.34
	20	120	4.22	15	125	7.16	33	88	5.8
	25	130	2.8	30	130	4.5	37	82	5.8
	30	135	1.68	20	107	7.16	22	90	7.78
Ripening Stage	17	120	2.8	25	135	9.57	32	107	7.73
	27	135	2.8	20	140	9.39	34	117	7.72
	15	135	4.3	30	151	4.5	38	127	5.8
	10	120	3	17	133	4.5	26	112	5.8
	30	140	1.68	23	159	7.16	23	111	5.8
Whole Growing Season	Temp. (°C)	Rainfall (mm)	Yield (t ha^− 1^)	Temp. (°C)	Rainfall (mm)	Yield (t ha^− 1^)	Temp. (°C)	Rainfall (mm)	Yield (t ha^− 1^)
	25	750	4.3	19	150	4.5	27	500	7.75
	30	1000	1.67	22	700	9.58	32	700	7.73
	20	500	4.22	25	299	9.53	37	1000	6
	17	300	1.71	30	800	4.5	21	900	7.75
	22	800	4.27	35	1000	7	30	200	4

Winter wheat emerges best between 5 to 7 days after sowing (DAS), winter maize between 12 to  17 DAS, and summer maize between 6 to  10 DAS. Both crops have optimum temperature ranges, and deliver better yields within these limits. In case the temperature goes beyond their optimum range, yield will surely be hampered. Similar trends show in case of winter and summer maize (Table 3). Similarly, during the flowering stage, wheat and maize yield will be high when the temperature lies within the optimum range and the days after sowing (DAS) are optimal. Using a fuzzy logic model, if the temperature decreases or increases with high variations from this range, then the yield will be reduced. For instance, at 10 °C and 30 °C temperature, the yield of winter wheat will be 2.8 tons/ha and 1.68 tons/ha, respectively (Table 3).

During the grain formation stage for winter wheat, the optimum temperature is below 25 °C, and the favorable time for grain formation is 105–110 days after sowing (DAS). We found that the yield of winter wheat will decrease significantly with an increase in temperature. On the other hand, for winter maize, temperatures near their optimum range will help achieve medium or high yield, but when it exceeds the optimum range, it shows low yield (Table 3). In the case of summer maize, grain formation occurs within 75–80 DAS, and the suitable temperature for grain formation is below 28 °C. Here, the fuzzy logic approach indicated that yield will be high when the temperature is below 28 °C and medium yield when the temperature is above 30 °C (Table 3).

**Fig. 10:**
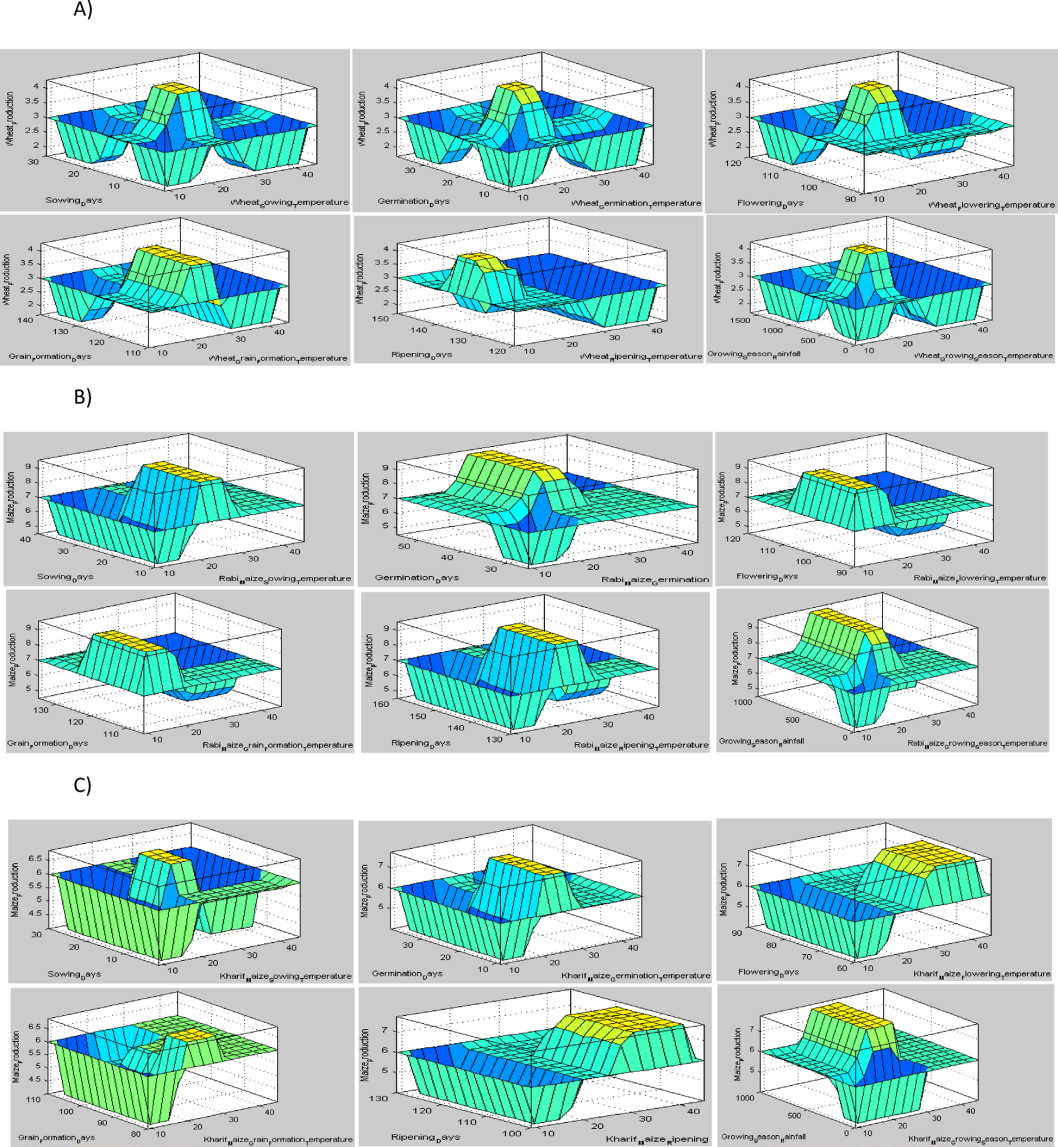
Surface view (3D) of wheat (**A**), winter (**B**) and summer (**C**) season maize yield prediction based on phenological stages and whole growing season; from left to right-sowing time, germination, flowering, grain formation, ripening stage, and whole growing season for each case by Fuzzy modelling system.

During the ripening stage, if the temperature is lower or higher than the optimum range for wheat and maize crops, yield will be low. From the experiment, we also estimated the proper rainfall and temperature effects on wheat and maize crop yield by using a fuzzy logic model. In case of wheat, the suitable temperature for highest yield is 21–26 °C, and the favorable rainfall during this time is 400–1100 mm. It showed that at high and low temperatures outside this range, the yield will be reduced. Similarly, the yield will be decreased with a shortage of rainfall. The growing season of winter maize requires a temperature range of 21 to 27 °C whereas the temperature range for summer maize range is 25–30 °C and both require rainfall ranges form 500–900 mm. We found that rainfall has a lower effect than temperature on the growing season of winter maize (Table 3), whereas rainfall has significant effects on summer maize crop yield. The surface view of projected result from the fuzzy system are shown in (Fig. 10).

### Estimation of optimum sowing window for wheat and maize crops

Sowing time is very crucial for getting optimum yield for both wheat and maize crops. Estimation of optimum sowing time assessed for wheat and maize based on the 24 years climate data (Table 4). Since the optimum temperature for wheat production during sowing time is 22–27 °C as well as germination and reproductive stage require 20–25 °C^121^ and 15–20 °C^63^respectively. Therefore, any delay of sowing time from November 1–7, will reduce the yield of wheat (Table 3), as beyond this limit the temperature goes outside the optimum range for proper germination and grain formation (Table 4). In case of winter maize, optimum sowing time is 1–10 November because outside of this range the seeds of winter maize can’t get proper temperature for germination and grain formation also will be hampered. As optimum temperature for germination (and early growth stages) and grain formation is 23–29 °C and 18–20 °C^64^ respectively. For instance, in December average temperature is about 18.37 °C which is very low than optimum range of temperature required for potential germination and early growth development (Table 4). Whereas for summer maize, suitable temperature for germination and early growth stages of maize seed is 20–28 °C and less than 28 °C temperature is favorable for grain formation stages (even temperature > 28 °C reduces yield by 10% yield). As grain formation begins 70–80 DAS, therefore, optimum planting time should be Feb 20 − 7 March which facilitates proper germination, early growth development and reproductive phases weather’s conditions (Table 4).

**Table 4:** Monthly weather data of Rajshahi (20 years mean).

	Average temperature (°C)	Rainfall (mm)
January	16.70	9.32
February	20.14	24.37
March	25.47	36.95
April	29.17	62.89
May	29.52	152.57
June	30.23	180.31
July	29.83	243.53
August	29.92	224.47
September	29.62	194.57
October	27.38	100.26
November	23.15	18.57
December	18.60	7.84

## Discussion

### Climate induced yield variability in maize and wheat crops

This study evaluates the impact of climate change on two important cereal crops (wheat and maize) in Rajshahi. The result demonstrated that temperature and rainfall have a influence on wheat and maize yield. Although, the influence of extreme temperature didn’t show any upward or downward trends, however, it had clear negative impact on summer maize yield which caused lower yield over the periods with small variations. On the other hand, precipitation showed a direct relation with yield which coincides with the findings of^122^ where winter wheat yield increased in almost all studied year. These findings align with^123^who stated that winter wheat yield grew by an average of 85.6 ± 15.1 kg ha^− 1^ per year. In this region, winter maize yield is always higher than the summer maize due to the favorable temperatures ranges throughout the winter maize season. Singh et al., 2010 ^127^ also reported the same findings in India. Due to the variations in temperature and rainfall, crop growth and maturation times are directly affected by various biotic and abiotic stresses^125^. A recent study found that 30–50% of global agricultural productivity losses are caused by these biotic and abiotic stressors^126^. Overall, these factors will directly affect food security, food safety and human health.

By 2050, wheat yield is projected to decrease by 36 to 40% due to climate change, whereas maize will experience minor effects than other cereals^127^. Climate change significantly affects both wheat and maize phenology as well as yield while Poggi et al. (2022)^128^ and Cann et al. (2023)^129^ also reported similar findings. The timing of significant phenological event has changed due to climate change, leading to shortened maturity period and reduced crop quality in terms of protein, fiber, and sugar content^130]– [131^. The reproductive stages of both maize and wheat crops are more sensitive to temperature than the vegetative stage^132]– [133^.

### Significance of fuzzy logic modeling for prediction of temperature effects and rainfall variability

The fuzzy logic helps to predict yield at different temperatures ranges, phenological stages (DAS), and rainfall levels. It explains fluctuation in yield caused by changes in climatic variables and the timing of the growing season of the crops. Temperature and rainfall have a major influence on every growth stage of wheat and maize, consequently on yield. Asseng et al., 2011^137^ stated the same findings that throughout almost every developmental stage of the crop growth cycle, the impacts of high temperatures on growth and development are evident. The results of this study using fuzzy forecast showed that mid-November sown wheat will produce 4.25 t ha^− 1^ with a a temperature range of 20 °C to 22 °C (Table 3). Fuzzy logic demonstrates how variations in the combination of rainfall and temperature at particular phenological stage influence yield. This finding aligns with Tao et al. (2015)^135 ^they stated temperature changes predominantly influence wheat phenology. During grain filling stage, high temperatures (30 °C) can lead to lower wheat yield of 1.86 t ha^− 1^. ShouChen et al. (2018)^136^ reported the same result that high temperature during grain filing stage reduced wheat yield. According to the fuzzy result, during the growing season of winter maize, if the average rainfall is 700 mm and the temperature is 22 °C, the yield will be 9.58 t ha^− 1^. However, yield will be decreased to 4.5t ha^− 1^ when the average rainfall increases to 800 mm, and temperature rises to 30 °C (Table 3). The temperature has a clearer effect than the rainfall on different growing stages of wheat and maize. In fuzzy analysis, the yield of summer maize will be 7.75 t ha^− 1^ at an average rainfall of 500 mm, however, the yield will fall to 7.73 t ha^− 1^ despite an increase of rainfall although the rainfall increased to 700 mm during the growing season (Table 3). When considering the entire growing season of wheat, winter maize, and summer maize, fuzzy predicts the most logical output based on the given logic made from the previous data. Fuzzy logic model showed that rainfall has a greater impact on summer sown crop than winter maize. The forecast of fuzzy for the growing season of winter maize showed the yield will be 7 t ha^− 1^ and 4.5t ha^− 1^ at an average rainfall of 100 mm and 150 mm, respectively while summer maize is projected to yield 7.75t ha^− 1^ and 4t ha^− 1^ at an average rainfall of 900 mm and 200 mm respectively (Table 3). Each growth stage of wheat and maize requires favorable conditions for optimal development, particularly during the reproductive phase, to produce maximum grain.

### Adaptation strategies for sowing times of wheat and maize crops

To mitigate the problem of yield loss in wheat and maize induced by changes in climatic variables (temperature and rainfall variability), farmers need to adapt measure like the changes in sowing time to coincide with the climate. Agronomic strategies that assist farmers in effectively adjusting to climate shocks and reducing productivity loss are considered climate-smart strategies and adaptation. This study focused reconsidering the sowing time for winter wheat and both winter and summer sown maize crop using fuzzy logical modeling approach. The significance of choosing the ideal sowing time for increased production at no additional cost has been widely demonstrated worldwide^137^. The ideal planting time varies according to the local growing conditions and variety^138^. The optimum sowing time for winter wheat in Rajshahi is found as November 1–7. This finding is consistent with the finding of Chhokar et al. (2022)^138^ stated that early showing produced 16% higher grain yield than timely showing in mid-November. In contrast, Jahan et al. (2018)^139^ reported that optimum showing time for wheat in Bangladesh is 15–30 November based on the weather data of Gazipur district which belongs from different climate and soil condition compared to Rajshahi. In case of winter maize, ideal sowing time is November 1–10. Similar findings were reported by Choudhury et al., (2021)^140^ and they reported optimum showing window for winter maize yield from 5 to 20 November in Jashore district (southwestern part of Bangaldesh). However, recommended sowing window for summer maize is February 20- March 7, which is harmonic with the government record (mid-February- March) as a general guideline for the whole country^141^.

The significance of these measures is to lessen the negative effects of temperature extremes^142^. However, adjusting the planting time may also assist in avoiding heat stress during the critical stage of growth^143^. Many of the negative effects of climate change can be addressed through future research and plant breeding programs. However, if farmers continue to grow the same crop varieties using the same practices in the same locations, then the projected detrimental effects of climate change will persist. In this study, we didn’t assess the impact of management factors on wheat and maize yield which is also very important for the yield variation. Thus, there is an opportunity to work on those factors to give more precise prediction through fuzzy logic.

Future research can be conducted under controlled environmental conditions that will provide more precise data to understand the subtle effects of climate change on the growth and development and consequently the yield of wheat and maize cultivated in northwestern Bangladesh. More research and well-structured government policies for this area could assist in dealing with the problems that arise from climate change.

### Relationship with SDGs

The 2030 Agenda for Sustainable Development, approved by all United Nations Member States in 2015, lays out a shared vision for peace and prosperity for people and the planet, both now and in the future. The 17 Sustainable Development Goals (SDGs) are at the heart of this agenda, representing an urgent call to action for all countries-, to work together globally (United Nations). As a member state of the UN, the People Republic of Bangladesh is striving to implement the SDGs by 2030. Therefore, our research work supports government policy aimed at achieving the SDGs. The study investigates the potential impacts of climate change on crop yields in northwestern Bangladesh, particularly focusing on wheat and maize yield. A fuzzy logic system was used to predict how the future climate variables, such as temperature and rainfall, affect crop yields in this region. By identifying these potential implications, the research directly contributes to several Sustainable Development Goals (SDGs) (Table 5). The findings of this study provide valuable insights for policymakers and agricultural stakeholders in Northwestern Bangladesh as they develop climate-resilient agriculture strategies, contributing to the broader agenda of sustainable development.

**Table 5:** Study relationship with SDGs .

SDG Number	Sustainable Development Goal	Relevance to the Study
SDG 2	Zero Hunger	The study evaluates the possible impact of climate change on food security by studying changes in wheat and maize yield, which are essential staples for local food supply.
SDG 13	Climate Action	The research contributes to attempts to minimize and adapt to climate change impacts in agricultural systems by predicting their consequences using fuzzy logic and recommended suitable planting time to mitigate loss due to climate change.
SDG 15	Life on Land	For climate change adaptation, agronomic practices like changing planting time, heat tolerant cultivars, tillage, and mulching should be employed for sustainable land use and conservation of biodiversity in the crop field like wheat and maize.

## Conclusion

Climate change significantly influences all growth stages of wheat and maize (winter and summer seasons), ultimately affecting yields. Prolonged exposure to elevated temperature and drought, particularly during flowering and grain filling stages, imposes significant stress on wheat and maize yield. Variations in temperature and rainfall subtly influence the phenology of wheat and maize. Temperature variations had a more pronounced effect on wheat and maize phenology than rainfall, though rainfall has a great impact on the growing season of summer maize compared to winter maize and wheat crops. Remarkably, maize yields in the winter season are consistently higher than in summer due to the detrimental effects of high summer temperatures on critical crop growth stages. Each development stage of wheat and maize requires optimal climatic conditions to produce maximum productivity; even minor deviation from these optimal ranges can lead to a significant yield reduction for both wheat and maize crops. Assessing and implementing adaptive strategies can mitigate yield losses under changing climatic conditions. To minimize climate related impacts, the recommended planting windows are 1–7 November for wheat, 1–10 November for winter maize and 20 February - 7 March for summer maize. These findings provide a valuable reference for further research and inform policymaking aligned with the Sustainable Development Goals (SDGs).

## Electronic supplementary material

Below is the link to the electronic supplementary material.

Supplementary Material 1

## Data Availability

Data will be made available on request from the first author and corresponding authors.
