# Supplementary material for: Modeling impacts of climate-induced yield variability and adaptations on wheat and maize in a sub-tropical monsoon climate - using fuzzy logic
Source: Sci Rep. 2025 Jul 16;15:25882. doi: 10.1038/s41598-025-09820-3 (PMC12267517; doi:10.1038/s41598-025-09820-3)
Supplement: Supplementary file 1 — Supplementary Material 1 [file 41598_2025_9820_MOESM1_ESM.docx]

**Modeling impacts of climate-induced yield variability and adaptations on wheat and maize in a sub-tropical monsoon climate - using fuzzy logic**

Md. Abdul Kaium^a,b1,^ , Md. Sharif Ahmed^a,b1,^, Muhammad Habib-ur-Rahman^c*^, Md. Saidul Islam^a,b^, Yeasmin Akter Ratry^d^, Md Mostofa Uddin Helal^e^, Muhammad Ali Fardoush Siddquy^f^, Most. Moslema Haque^a^, Ahsan Raza^g,h*^, Fatma Mansour^i^ , Majed Alotaibi^j^, Ayman El Sabagh^k^, Reimund P. Roetter ^c,l^

^a^Department of Crop Science and Technology, University of Rajshahi, Rajshahi 6205, Bangladesh

^b^Institute of Natural Resources Research and Development, Rajshahi 6206, Bangladesh

^c^ Tropical Plant Production and Agricultural Systems Modelling (TROPAGS), University of Goettingen, Grisebachstr. 6, 37077, Goettingen, Germany

^d^Department of Mathematics, University of Rajshahi, Rajshahi 6205, Bangladesh

^e^State Key Laboratory of Sustainable Dryland Agriculture, Institute of Wheat Research, College of Agriculture, Shanxi Agricultural University, Linfen 041000, Shanxi, China

^f^Graduate School of Bioresources, Mie University, 1577 Kurimamachiya-Cho, Tsu, Mie, 514-8507, Japan

^g^ Crop Science, Institute of Crop Science and Resource Conservation (INRES), University of Bonn, 53115, Bonn, Germany

^h^ Leibniz Centre for Agricultural Landscape Research (ZALF), Eberswalder Straße 84, 15374, Müncheberg, Germany

^i^Department of Economics, Business and Economics Faculty, Siirt University, Siirt, Turkey

^j^Plant Production Department, College of Food and Agricultural Sciences, King Saud University, P.O. Box. 2460, Riyadh 11451, Saudi Arabia

^k^Siirt University, Faculty of Agriculture, Department of Field Crops, 56100 Siirt, Turkey

^l^Campus Centre of Biodiversity and Sustainable Land Use (CBL), University of Goettingen, Buesgenweg 1, 37077, Goettingen, Germany

^1^ Consider as first author

*Corresponding authors E-mail address: [habib.rahman@uni-goettingen.de](mailto:habib.rahman@uni-goettingen.de) and [ahsan.raza@zalf.de](mailto:ahsan.raza@zalf.de); araza@uni-bonn.de

**Table S1: Fuzzy linguistic variables and their membership functions for temperature, rainfall and production**

| Production | | | | | | | | | |
| --- | --- | --- | --- | --- | --- | --- | --- | --- | --- |
| Wheat (ton/ha) | | | | Rabi maize (ton/ha) | | | Kharif maize (ton/ha) | | |
|  | Lower V. | Mid  V. | Upper V. | Lower  V. | Mid  V. | Upper V. | Lower V. | Mid  V. | Upper  V. |
| Low | 1 | 1.9 | 2.3 | 3 | 4.5 | 6 | 3 | 4 | 5 |
| Average | 2.1 | 2.8 | 3.5 | 5.8 | 7.2 | 8.5 | 4.8 | 5.8 | 6.8 |
| High | 3.4 | 4.5 | 5 | 8.2 | 9.5 | 11 | 6.6 | 7.5 | 9 |

*V indicates value

**Table S2: Yield of wheat among Rajshahi, Bangladesh and Global (2000-2023)**

| year | Rajshahi | Bangladesh | Global |
| --- | --- | --- | --- |
| 2000 | 2.30 | 2.21 | 2.73 |
| 2001 | 2.14 | 2.16 | 2.74 |
| 2002 | 2.21 | 2.16 | 2.76 |
| 2003 | 2.45 | 2.13 | 2.65 |
| 2004 | 2.11 | 1.95 | 2.94 |
| 2005 | 1.59 | 1.75 | 2.83 |
| 2006 | 2.35 | 1.53 | 2.89 |
| 2007 | 3.18 | 1.85 | 2.82 |
| 2008 | 2.50 | 2.18 | 3.06 |
| 2009 | 2.80 | 2.40 | 3.04 |
| 2010 | 2.92 | 2.60 | 2.97 |
| 2011 | 3.32 | 2.78 | 3.16 |
| 2012 | 3.55 | 3.01 | 3.09 |
| 2013 | 3.60 | 3.03 | 3.25 |
| 2014 | 3.50 | 3.09 | 3.32 |
| 2015 | 2.78 | 3.03 | 3.32 |
| 2016 | 3.26 | 3.16 | 3.42 |
| 2017 | 3.13 | 3.13 | 3.54 |
| 2018 | 3.56 | 3.08 | 3.42 |
| 2019 | 3.56 | 3.10 | 3.54 |
| 2020 | 3.60 | 3.30 | 3.47 |
| 2021 | 3.89 | 3.45 | 3.49 |
| 2022 | 3.23 | 3.45 | 3.70 |
| 2023 | 3.38 | 3.69 | 3.62 |
| Mean | 2.92 | 2.74 | 3.15 |
| SD | 0.64 | 0.62 | 0.32 |
| Maximum | 3.89 | 3.69 | 3.70 |
| Minimum | 1.58 | 1.53 | 2.65 |

Data Sources: Bangladesh Bureau of Statistics (BBS), Department of Agricultural Extension (DAE), Rajshahi; FAOstat

**Table S3: Yield of maize among Rajshahi, Bangladesh and World (2000-2023)**

| year | Rajshahi | Bangladesh | Global |
| --- | --- | --- | --- |
| 2000 | 3.27 | 2.06 | 4.32 |
| 2001 | 3.39 | 3.22 | 4.48 |
| 2002 | 4.19 | 4.03 | 4.39 |
| 2003 | 3.89 | 4.03 | 4.46 |
| 2004 | 4.08 | 4.82 | 4.95 |
| 2005 | 4.79 | 5.33 | 4.82 |
| 2006 | 4.69 | 5.30 | 4.78 |
| 2007 | 4.92 | 5.98 | 4.98 |
| 2008 | 4.65 | 6.02 | 5.07 |
| 2009 | 4.83 | 5.83 | 5.15 |
| 2010 | 5.21 | 6.15 | 5.16 |
| 2011 | 5.67 | 6.58 | 5.14 |
| 2012 | 5.99 | 6.59 | 4.85 |
| 2013 | 6.60 | 6.91 | 5.42 |
| 2014 | 6.30 | 6.98 | 5.58 |
| 2015 | 6.15 | 7.30 | 5.52 |
| 2016 | 6.56 | 7.76 | 5.78 |
| 2017 | 6.87 | 8.20 | 5.74 |
| 2018 | 7.41 | 8.01 | 5.75 |
| 2019 | 8.45 | 8.51 | 5.85 |
| 2020 | 8.53 | 8.57 | 5.82 |
| 2021 | 8.86 | 8.91 | 5.88 |
| 2022 | 7.32 | 8.91 | 5.71 |
| 2023 | 7.61 | 9.25 | 5.96 |
| Mean | 5.84 | 6.67 | 5.23 |
| SD | 1.63 | 1.92 | 0.52 |
| Maximum | 8.86 | 9.25 | 5.96 |
| Minimum | 3.27 | 2.05 | 4.32 |

Data Source: Bangladesh Bureau of Statistics (BBS), Department of Agricultural Extension (DAE), Rajshahi; FAOstat

**Table S4: Wheat yield comparison with top growing countries (2000-2023)**

| year | Rajshahi | Bangladesh | Global | China | India | Russia | USA | France | Ukraine | Australia | Pakistan | Canada | Germany |
| --- | --- | --- | --- | --- | --- | --- | --- | --- | --- | --- | --- | --- | --- |
| 2000 | 2.30 | 2.21 | 2.73 | 3.73 | 2.77 | 1.61 | 2.82 | 7.11 | 1.97 | 2.00 | 2.49 | 2.44 | 7.28 |
| 2001 | 2.14 | 2.16 | 2.74 | 3.80 | 2.70 | 2.05 | 2.70 | 6.61 | 3.10 | 1.82 | 2.32 | 1.94 | 7.88 |
| 2002 | 2.21 | 2.16 | 2.76 | 3.77 | 2.76 | 2.06 | 2.35 | 7.44 | 3.04 | 2.10 | 2.26 | 1.83 | 6.90 |
| 2003 | 2.45 | 2.13 | 2.65 | 3.93 | 2.61 | 1.70 | 2.97 | 6.25 | 2.93 | 0.90 | 2.38 | 2.25 | 6.49 |
| 2004 | 2.11 | 1.95 | 2.94 | 4.25 | 2.71 | 1.98 | 2.90 | 7.57 | 3.16 | 1.99 | 2.37 | 2.64 | 8.17 |
| 2005 | 1.59 | 1.75 | 2.83 | 4.27 | 2.60 | 1.92 | 2.82 | 6.98 | 2.84 | 1.63 | 2.58 | 2.73 | 7.46 |
| 2006 | 2.35 | 1.53 | 2.89 | 4.59 | 2.61 | 1.94 | 2.59 | 6.74 | 2.53 | 2.02 | 2.51 | 2.60 | 7.20 |
| 2007 | 3.18 | 1.85 | 2.82 | 4.60 | 2.70 | 2.10 | 2.70 | 6.25 | 2.34 | 0.91 | 2.71 | 2.33 | 6.96 |
| 2008 | 2.50 | 2.18 | 3.06 | 4.76 | 2.80 | 2.44 | 3.01 | 7.10 | 3.66 | 1.07 | 2.45 | 2.85 | 8.08 |
| 2009 | 2.80 | 2.40 | 3.04 | 4.74 | 2.91 | 2.32 | 2.99 | 7.45 | 3.09 | 1.58 | 2.66 | 2.79 | 7.81 |
| 2010 | 2.92 | 2.60 | 2.97 | 4.75 | 2.84 | 1.92 | 3.12 | 7.04 | 2.68 | 1.57 | 2.55 | 2.81 | 7.21 |
| 2011 | 3.32 | 2.78 | 3.16 | 4.84 | 2.99 | 2.26 | 2.94 | 6.66 | 3.35 | 2.03 | 2.83 | 2.96 | 7.01 |
| 2012 | 3.55 | 3.01 | 3.09 | 4.99 | 3.18 | 1.77 | 3.12 | 7.15 | 2.80 | 2.15 | 2.71 | 2.87 | 7.33 |
| 2013 | 3.60 | 3.03 | 3.25 | 5.06 | 3.15 | 2.23 | 3.17 | 7.27 | 3.39 | 1.76 | 2.80 | 3.60 | 8.00 |
| 2014 | 3.50 | 3.09 | 3.32 | 5.24 | 3.15 | 2.50 | 2.94 | 7.35 | 4.01 | 2.01 | 2.82 | 3.08 | 8.63 |
| 2015 | 2.78 | 3.03 | 3.32 | 5.39 | 2.75 | 2.39 | 2.93 | 7.80 | 3.88 | 1.92 | 2.73 | 2.89 | 8.09 |
| 2016 | 3.26 | 3.16 | 3.42 | 5.40 | 3.03 | 2.69 | 3.54 | 5.29 | 4.21 | 1.97 | 2.78 | 3.58 | 7.64 |
| 2017 | 3.13 | 3.13 | 3.54 | 5.48 | 3.20 | 3.13 | 3.12 | 7.25 | 4.11 | 2.61 | 2.97 | 3.38 | 7.64 |
| 2018 | 3.56 | 3.08 | 3.42 | 5.42 | 3.37 | 2.72 | 3.20 | 6.77 | 3.72 | 1.92 | 2.85 | 3.27 | 6.67 |
| 2019 | 3.56 | 3.10 | 3.54 | 5.63 | 3.53 | 2.70 | 3.47 | 7.74 | 4.16 | 1.69 | 2.81 | 3.38 | 7.40 |
| 2020 | 3.60 | 3.30 | 3.47 | 5.74 | 3.44 | 2.98 | 3.34 | 6.68 | 3.80 | 1.47 | 2.87 | 3.54 | 7.82 |
| 2021 | 3.89 | 3.45 | 3.49 | 5.81 | 3.47 | 2.72 | 2.98 | 6.93 | 4.53 | 2.52 | 3.00 | 2.41 | 7.30 |
| 2022 | 3.23 | 3.45 | 3.70 | 5.85 | 3.53 | 3.55 | 3.12 | 6.99 | 3.92 | 2.84 | 2.99 | 3.40 | 7.57 |
| 2023 | 3.38 | 3.69 | 3.62 | 5.78 | 3.52 | 3.17 | 3.26 | 7.20 | 4.64 | 3.18 | 3.11 | 2.99 | 7.43 |

Data Source: Bangladesh Bureau of Statistics (BBS), Department of Agricultural Extension (DAE), Rajshahi; FAOstat

**Table S5: Maize yield comparison with top growing countries (2000-2023)**

| year | Rajshahi | Bangladesh | Global | USA | Chaina | Brazil | Argentina | Ukraine | India | Mexico | Indonesia | South Africa | France |
| --- | --- | --- | --- | --- | --- | --- | --- | --- | --- | --- | --- | --- | --- |
| 2000 | 3.27 | 2.06 | 4.32 | 8.59 | 4.59 | 2.71 | 5.43 | 3.00 | 1.82 | 2.46 | 2.76 | 2.84 | 9.07 |
| 2001 | 3.39 | 3.22 | 4.48 | 8.67 | 4.69 | 3.40 | 5.45 | 3.24 | 1.99 | 2.57 | 2.84 | 2.43 | 8.57 |
| 2002 | 4.19 | 4.03 | 4.39 | 8.11 | 4.87 | 3.05 | 6.07 | 3.51 | 1.68 | 2.71 | 3.06 | 2.85 | 8.97 |
| 2003 | 3.89 | 4.03 | 4.46 | 8.92 | 4.81 | 3.72 | 6.47 | 3.45 | 2.04 | 2.75 | 3.24 | 2.65 | 7.15 |
| 2004 | 4.08 | 4.82 | 4.95 | 10.06 | 5.12 | 3.36 | 6.39 | 3.85 | 1.90 | 2.81 | 3.34 | 3.03 | 8.99 |
| 2005 | 4.79 | 5.33 | 4.82 | 9.28 | 5.28 | 3.04 | 7.35 | 4.31 | 1.93 | 2.92 | 3.45 | 3.63 | 8.25 |
| 2006 | 4.69 | 5.30 | 4.78 | 9.35 | 5.32 | 3.38 | 5.90 | 3.73 | 1.91 | 3.00 | 3.46 | 3.41 | 8.58 |
| 2007 | 4.92 | 5.98 | 4.98 | 9.45 | 5.16 | 3.78 | 7.66 | 3.90 | 2.33 | 3.20 | 3.66 | 2.79 | 9.48 |
| 2008 | 4.65 | 6.02 | 5.07 | 9.62 | 5.55 | 4.08 | 6.45 | 4.69 | 2.41 | 3.30 | 4.07 | 4.53 | 9.12 |
| 2009 | 4.83 | 5.83 | 5.15 | 10.32 | 5.26 | 3.71 | 5.58 | 5.02 | 2.02 | 3.24 | 4.24 | 4.96 | 8.89 |
| 2010 | 5.21 | 6.15 | 5.16 | 9.58 | 5.46 | 4.37 | 7.80 | 4.51 | 2.54 | 3.26 | 4.44 | 4.67 | 8.83 |
| 2011 | 5.67 | 6.58 | 5.14 | 9.21 | 5.75 | 4.21 | 6.35 | 6.44 | 2.48 | 2.91 | 4.57 | 4.37 | 9.97 |
| 2012 | 5.99 | 6.59 | 4.85 | 7.73 | 5.87 | 5.01 | 5.73 | 4.79 | 2.56 | 3.19 | 4.90 | 4.49 | 9.00 |
| 2013 | 6.60 | 6.91 | 5.42 | 9.93 | 6.02 | 5.25 | 6.60 | 6.41 | 2.57 | 3.19 | 4.84 | 4.25 | 8.16 |
| 2014 | 6.30 | 6.98 | 5.58 | 10.73 | 5.81 | 5.18 | 6.84 | 6.16 | 2.57 | 3.30 | 4.95 | 5.30 | 10.05 |
| 2015 | 6.15 | 7.30 | 5.52 | 10.57 | 5.89 | 5.54 | 7.31 | 5.71 | 2.61 | 3.48 | 5.18 | 3.49 | 8.38 |
| 2016 | 6.56 | 7.76 | 5.78 | 11.74 | 5.97 | 4.29 | 7.44 | 6.60 | 2.60 | 3.72 | 5.31 | 3.71 | 8.21 |
| 2017 | 6.87 | 8.20 | 5.74 | 11.08 | 6.11 | 5.62 | 7.58 | 5.51 | 2.69 | 3.79 | 5.23 | 5.86 | 10.12 |
| 2018 | 7.41 | 8.01 | 5.75 | 11.07 | 6.10 | 5.11 | 6.09 | 7.84 | 3.07 | 3.81 | 5.33 | 4.98 | 8.82 |
| 2019 | 8.45 | 8.51 | 5.85 | 10.51 | 6.32 | 5.77 | 7.86 | 7.19 | 3.07 | 4.07 | 5.45 | 4.55 | 8.53 |
| 2020 | 8.53 | 8.57 | 5.82 | 10.76 | 6.32 | 5.70 | 7.55 | 5.62 | 3.01 | 3.83 | 5.70 | 5.45 | 7.94 |
| 2021 | 8.86 | 8.91 | 5.88 | 11.11 | 6.29 | 4.65 | 7.43 | 7.68 | 3.21 | 3.85 | 5.72 | 5.41 | 9.91 |
| 2022 | 7.32 | 8.91 | 5.71 | 10.88 | 6.43 | 5.20 | 6.73 | 6.34 | 3.38 | 3.89 | 8.08 | 5.89 | 7.47 |
| 2023 | 7.61 | 9.25 | 5.96 | 11.13 | 6.53 | 5.91 | 5.10 | 7.80 | 3.54 | 4.28 | 8.07 | 6.35 | 9.76 |

Data Source: Bangladesh Bureau of Statistics (BBS), Department of Agricultural Extension (DAE), Rajshahi; FAOstat

**Table S6: Monthly average rainfall (mm) of Rajshahi from 2000-2024**

| Year | Jul | Aug | Sep | Oct | Nov | Dec | Jan | Feb | Mar | Apr | May | Jun |
| --- | --- | --- | --- | --- | --- | --- | --- | --- | --- | --- | --- | --- |
| 2000-2001 | 338 | 209 | 95 | 184 | 1 | 0 | 0 | 0 | 9 | 13 | 209 | 324 |
| 2001-2002 | 316 | 238 | 281 | 48 | 17 | 0 | 10 | 1 | 20 | 96 | 196 | 222 |
| 2002-2003 | 230 | 128 | 262 | 292 | 0 | 6 | 3 | 18 | 64 | 45 | 84 | 280 |
| 2003-2004 | 339 | 275 | 449 | 153 | 0 | 0 | 10 | 0 | 0 | 61 | 92 | 507 |
| 2004-2005 | 492 | 161 | 131 | 275 | 0 | 0 | 14 | 1 | 104 | 27 | 108 | 92 |
| 2005-2006 | 130 | 247 | 302 | 36 | 10 | 0 | 0 | 0 | 7 | 36 | 189 | 188 |
| 2006-2007 | 364 | 236 | 309 | 76 | 1 | 0 | 0 | 27 | 59 | 13 | 260 | 313 |
| 2007-2008 | 373 | 245 | 129 | 121 | 0 | 0 | 26 | 0 | 0 | 30 | 144 | 247 |
| 2008-2009 | 183 | 240 | 282 | 45 | 0 | 0 | 1 | 7 | 28 | 0 | 131 | 126 |
| 2009-2010 | 183 | 240 | 282 | 45 | 0 | 0 | 1 | 2 | 2 | 37 | 75 | 211 |
| 2010-2011 | 94 | 101 | 101 | 127 | 3 | 39 | 6 | 0 | 10 | 94 | 187 | 341 |
| 2011-2012 | 144 | 554 | 203 | 35 | 1 | 0 | 6 | 0 | 6 | 123 | 17 | 137 |
| 2012-2013 | 314 | 179 | 178 | 102 | 101 | 1 | 0 | 22 | 12 | 51 | 188 | 178 |
| 2013-2014 | 101 | 254 | 238 | 204 | 0 | 0 | 0 | 27 | 12 | 51 | 151 | 188 |
| 2014-2015 | 242 | 359 | 153 | 5 | 0 | 0 | 14 | 14 | 39 | 144 | 177 | 285 |
| 2015-2016 | 353 | 127 | 254 | 7 | 6 | 1 | 42 | 3 | 25 | 175 | 212 | 109 |
| 2016-2017 | 376 | 168 | 170 | 95 | 0 | 0 | 2 | 0 | 45 | 80 | 193 | 141 |
| 2017-2018 | 393 | 215 | 132 | 237 | 5 | 20 | 0 | 12 | 9 | 75 | 176 | 139 |
| 2018-2019 | 238 | 84 | 162 | 87 | 0 | 17 | 0 | 46 | 67 | 114 | 147 | 121 |
| 2019-2020 | 262 | 114 | 187 | 174 | 3 | 0 | 16 | 1 | 27 | 32 | 187 | 283 |
| 2020-2021 | 412 | 142 | 213 | 98 | 0 | 0 | 0 | 0 | 0 | 15 | 140 | 263 |
| 2021-2022 | 325 | 542 | 135 | 116 | 1 | 1 | 3 | 39 | 0 | 20 | 222 | 70 |
| 2022-2023 | 3 | 39 | 0 | 20 | 222 | 70 | 60 | 263 | 329 | 57 | 0 | 1 |
| 2023-2024 | 137 | 179 | 267 | 275 | 0 | 0 | 0 | 0 | 25 | 48 | 103 | 85 |

Source: Bangladesh Meteorological Department

**Table S7: Monthly average maximum temperature (°C) of Rajshahi from 2000-2024**

| Year | Jul | Aug | Sep | Oct | Nov | Dec | Jan | Feb | Mar | Apri | May | Jun |
| --- | --- | --- | --- | --- | --- | --- | --- | --- | --- | --- | --- | --- |
| 2000-2001 | 32.7 | 33.3 | 31.2 | 32.1 | 30.2 | 26.1 | 24.2 | 28.6 | 33.6 | 37 | 33 | 32.5 |
| 2001-2002 | 32.7 | 33.5 | 33.2 | 31.9 | 29.7 | 25 | 25 | 28.7 | 33.2 | 33.5 | 32.9 | 33.5 |
| 2002-2003 | 33.2 | 32.6 | 33.1 | 31.9 | 29.1 | 25.5 | 21.8 | 27.4 | 30.7 |  | 35.8 | 33.5 |
| 2003-2004 | 33.2 | 33.7 | 32.8 | 30.7 | 29.4 | 25.6 | 22 | 28.1 | 34.5 | 34.8 | 37.1 | 33.5 |
| 2004-2005 | 32.4 | 33.1 | 31.5 | 31.1 | 29.5 | 26.3 | 23.8 | 29.1 | 32.9 | 35.8 | 35.1 | 36 |
| 2005-2006 | 32.2 | 33.2 | 33.7 | 29.8 | 28.3 | 26.5 | 24.9 | 31.3 | 33.5 | 35.3 | 35 | 34.1 |
| 2006-2007 | 33.2 | 33.3 | 32.9 | 32.6 | 28.7 | 26.3 | 24.5 | 26.4 | 31.1 | 35.4 | 36.1 | 33.8 |
| 2007-2008 | 32.4 | 33.2 | 32.8 | 31.9 | 29.7 | 25.4 | 23.7 | 25.7 | 33 | 36.3 | 35.6 | 32.7 |
| 2008-2009 | 31.9 | 32.9 | 32.2 | 31.8 | 29.6 | 25 | 24.5 | 29.5 | 33.4 | 37.4 | 34.9 | 36.7 |
| 2009-2010 | 33.5 | 32.9 | 33.6 | 31.9 | 29.7 | 25.4 | 22.4 | 28.8 | 35.9 | 38.3 | 35.9 | 35 |
| 2010-2011 | 34 | 34 | 33.2 | 32 | 29.8 | 25.1 | 22.6 | 28.3 | 33.7 | 34.8 | 34.6 | 34.1 |
| 2011-2012 | 33.5 | 32.2 | 32.8 | 32.9 | 29.1 | 24.2 | 23.4 | 28.4 | 33.9 | 35.5 | 38.3 | 36.2 |
| 2012-2013 | 33.4 | 32.2 | 33.5 | 32.4 | 28.2 | 23.1 | 23.4 | 27.9 | 33.9 | 36.4 | 33.9 | 35.2 |
| 2013-2014 | 34.3 | 33.4 | 34.4 | 31.4 | 29.6 | 25.5 | 23 | 26 | 32.5 | 38 | 37.1 | 34.9 |
| 2014-2015 | 33.9 | 33.8 | 34 | 32.5 | 30.1 | 24.5 | 23.7 | 28.5 | 33 | 33.8 | 36 | 34.5 |
| 2015-2016 | 33.4 | 33.8 | 34.3 | 32.9 | 30.5 | 25.3 | 24.1 | 29.4 | 34 | 37.7 | 34.7 | 35.3 |
| 2016-2017 | 33.3 | 34.1 | 34 | 33 | 29.9 | 26.1 | 18.1 | 21.2 | 24.9 | 29.2 | 30 | 30.8 |
| 2017-2018 | 29.5 | 30.5 | 30.3 | 28.1 | 23.4 | 20.4 | 22.5 | 28.8 | 34.1 | 34 | 33.4 | 35.6 |
| 2018-2019 | 33.9 | 34.6 | 34.3 | 32.1 | 30 | 25.2 | 25.5 | 27.6 | 32.1 | 34.5 | 36.1 | 35.8 |
| 2019-2020 | 34.5 | 34.5 | 33.4 | 31.4 | 30 | 24.4 | 23.3 | 26.7 | 31.6 | 34.5 | 33.7 | 34.7 |
| 2020-2021 | 33.8 | 34.4 | 34 | 33.6 | 30.2 | 25.1 | 23.7 | 29 | 34.5 | 37.5 | 34.6 | 34.1 |
| 2021-2022 | 34.1 | 34.1 | 34.1 | 33.1 | 29.4 | 26.3 | 23.4 | 26 | 34.8 | 36.7 | 34.7 | 34.9 |
| 2022-2023 | 36.4 | 34.7 | 34 | 33 | 30.3 | 26.7 | 24.2 | 29.5 | 32.8 | 37.5 | 36.3 | 37.3 |
| 2023-2024 | 35.8 | 34 | 34.3 | 32.4 | 29.6 | 25.3 | 22 | 27.5 | 32.8 | 39 | 35.8 | 36.5 |

Source: Bangladesh Meteorological Department
